# hPCL3S promotes proliferation and migration of androgen-independent prostate cancer cells

**DOI:** 10.18632/oncotarget.27511

**Published:** 2020-03-24

**Authors:** Souhila Abdelfettah, Gaylor Boulay, Marion Dubuissez, Nathalie Spruyt, Sara P. Garcia, Shruthi Rengarajan, Ingrid Loison, Xavier Leroy, Miguel N. Rivera, Dominique Leprince

**Affiliations:** ^1^Department of Pathology, Center for Cancer Research, Massachusetts General Hospital and Harvard Medical School, Boston, MA 02114, USA; ^2^Department of Pathology, University de Lille, CHU de Lille, F-59000 Lille, France; ^3^Present Address: Maisonneuve-Rosemont Hospital Research Center, Maisonneuve-Rosemont Hospital, Montreal, QC H1T 3W5, Canada; ^4^University de Lille, CNRS, Institut Pasteur de Lille, UMR 8161m M3T, Mechanisms of Tumorigenesis and Targeted Therapies, F-59000 Lille, France

**Keywords:** hPCL3S, PHF19, PRC2, β-catenin, prostate cancer

## Abstract

Polycomb repressive complex 2 (PRC2) allows the deposition of H3K27me3. PRC2 facultative subunits modulate its activity and recruitment such as hPCL3/PHF19, a human ortholog of Drosophila Polycomb-like protein (PCL). These proteins contain a TUDOR domain binding H3K36me3, two PHD domains and a “Winged-helix” domain involved in GC-rich DNA binding. The human PCL3 locus encodes the full-length hPCL3L protein and a shorter isoform, hPCL3S containing the TUDOR and PHD1 domains only.

In this study, we demonstrated by RT-qPCR analyses of 25 prostate tumors that hPCL3S is frequently up-regulated. In addition, hPCL3S is overexpressed in the androgen-independent DU145 and PC3 cells, but not in the androgen-dependent LNCaP cells. *hPCL3S* knockdown decreased the proliferation and migration of DU145 and PC3 whereas its forced expression into LNCaP increased these properties. A mutant hPCL3S unable to bind H3K36me3 (TUDOR-W50A) increased proliferation and migration of LNCaP similarly to wt hPCL3S whereas inactivation of its PHD1 domain decreased proliferation. These effects partially relied on the up-regulation of genes known to be important for the proliferation and/or migration of prostate cancer cells such as *S100A16, PlexinA2*, and *Spondin1*.

Collectively, our results suggest hPCL3S as a new potential therapeutic target in castration resistant prostate cancers.

## INTRODUCTION

Prostate cancer (PCa) is the second most common type of cancer diagnosed worldwide and is still a leading cause of death despite recent research advances. The vast majority of PCa are classified as adenocarcinomas, a tumor type deriving from glandular cells of the prostate. Androgen and Androgen Receptor (AR) signaling are critical not only for the development and function of normal prostate but also for tumor development and are thus widely used as therapeutic targets. Initially, treatments with androgen depletion or AR blocking called androgen dependent therapy (ADT) are efficient. Unfortunately, however, most patients relapse and progress to androgen-independent more aggressive forms of prostate cancer termed castration-resistant prostate cancer (CRPC) with poor prognosis [1]. One of the mechanisms leading to this castration resistant disease is the manifestation of a neuroendocrine transdifferentiation (NE) following ADT and resulting in prostatic small cell neuroendocrine carcinomas (SCNC). These SCNC tumors cells fail to express PSA and AR but rather express neuroendocrine markers such as Neuron-specific Enolase (NES) or Chromogranin A (CgA) as well as stem-cell associated markers such as ALDH1A1 and CD44 [2].

Two of most commonly used prostate cancer cell lines LNCaP and PC3 are representative of prostatic adenocarcinoma and of Small Cell Neuroendocrine Carcinomas, respectively [3]. Neuroendocrine transdifferentiation of LNCaP cells can be achieved through the ectopic expression of component of the Wnt signaling pathway [4, 5] or activation of STAT3 [6].

**Figure 1:**
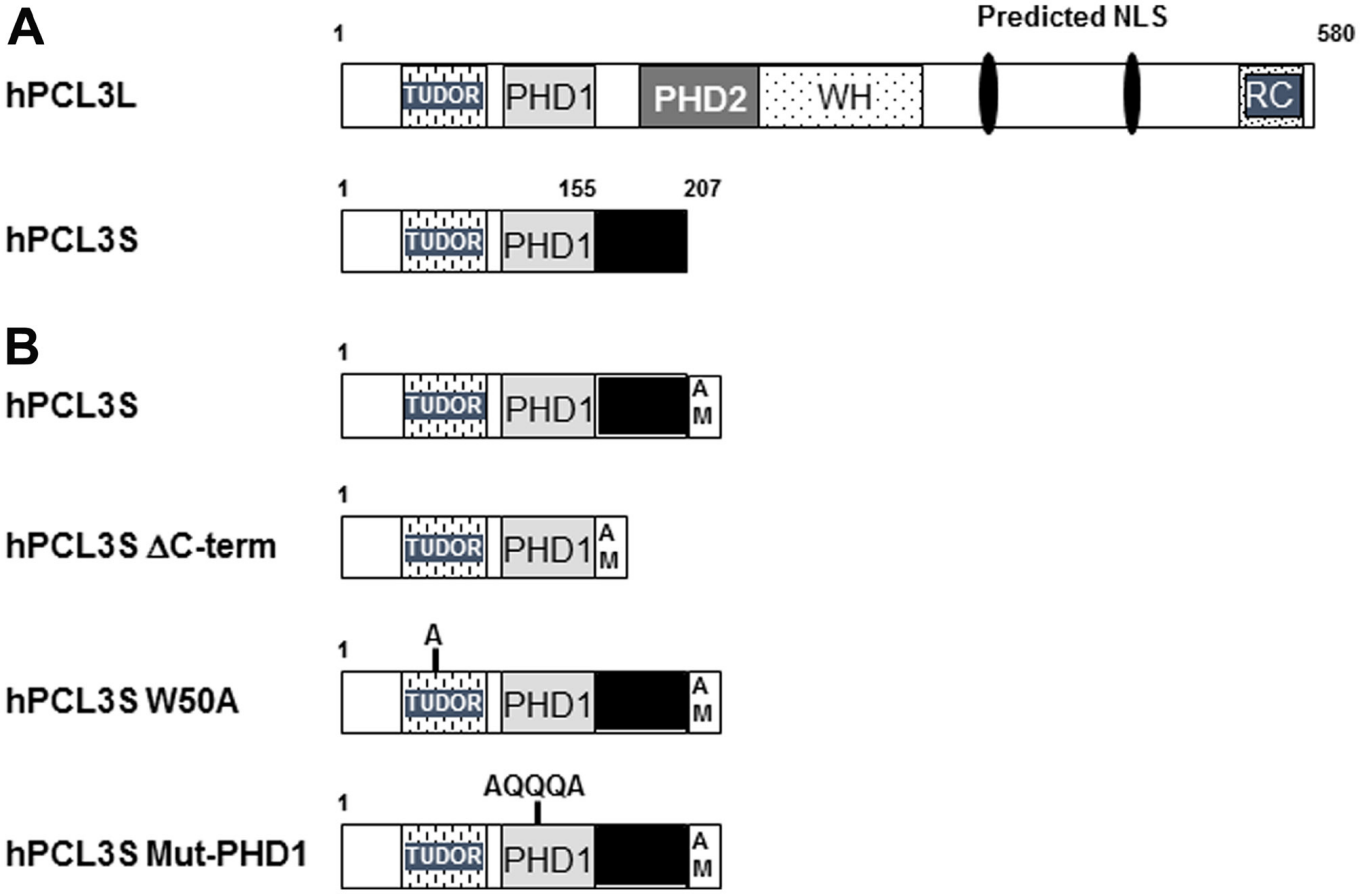
Human hPCL3L and hPCL3S isoforms and their identified functional domains. (**A**) Comparative structure of hPCL3L and hPCL3S. The structure of the full length human hPCL3L and of the shortest isoform hPCL3S generated by alternative splicing and Alternative Polyadenylation in the coding region (CR-APA) is schematically drawn [21, 22]. The various functional domains are indicated: the TUDOR domain; the two PHD (Plant Homeo domain); the Winged Helix domain involved in the binding to G-C-rich sequences [19, 20] and initially identified as an extended region of Homology (EH) with the Drosophila PCL protein [21] and finally the Reverse Chromodomain (RC) involved in the competitive interaction with AEBP2 for SUZ12 [15]. Two putative nuclear localization signals identified in hPCL3L are also indicated [21]. (**B**) Structure of the various hPCL3S constructs used in this study. Schematic structure of the wt hPCL3S-AMTag protein and mutants thereof (ΔC-term, W50A and Mut-PHD1). The C-terminal AM-Tag is shown as an open box.

The Polycomb repressive complex 2 (PRC2) is one of the two main multimeric complexes of Polycomb group of proteins (PcG), initially identified in Drosophila, which are crucial for development of multicellular organisms [7, 8]. PRC2 allows the deposition of the repressive epigenetic mark H3K27me3 by its catalytic subunit, *EZH2* [7, 9, 10]. Depending on the cancer type, *EZH2* is either overexpressed (prostate) or subject to loss or gain of function mutations, leading to aberrant levels of H3K27me3 [11, 12, 13]. *In vitro*, a tetramer consisting of the “core” PRC2 subunits, EZH2, SUZ12, EED, and RBBP4 is sufficient to catalyze the trimethylation of H3K27. However, *in vivo*, several facultative subunits modulating the enzymatic activity of the PRC2 complex or participating in its recruitment and/or its stabilization at target loci have been identified [10, 14]. They assemble with the core PRC2 subunits in mostly mutually exclusive combinations giving thus rise to two distinct classes of complexes, PRC2.1 and PRC2.2. PRC2.2 complexes are characterized by the AEBP2 and JARID2 facultative subunits. PRC2.1 complexes contain either of the two proteins EPOP or C10orf12 together with one of the three human orthologs, PHF1, PCL2 and hPCL3/PHF19 of the Drosophila Polycomb-like protein (PCL) [10, 14]. These proteins share a structured N-terminal domain consisting of a TUDOR domain, two PHD domains (Plant Homeo Domain) followed by a “Winged-helix” domain involved in GC-rich DNA binding and a C-terminal “reverse” Chromodomain (RC) (Figure 1A). This RC domain and a domain of AEBP2 which are in competition for the binding to the core component SUZ12 likely dictate the mutually exclusive association of hPCL3/PHF19 and AEBP2 in PRC2 complexes [15]. hPCL3/PHF19 regulates Polycomb PRC2 recruitment since it allows intrusion of PRC2 complexes into actively transcribed chromatin regions through binding to the active H3K36me3 epigenetic mark via an “aromatic cage” constituted by amino acids W50, Y56, F74, and Y80 in its TUDOR domain [16, 17, 18] and to GC-rich sequences [19, 20] (for review [10]). In addition, through its association with the H3K36 demethylase NOD66, hPCL3/PHF19 also favors the activation of EZH2 and H3K27me3 deposition [17].

Owing to different polyadenylation sites and alternative splicing events, the human *hPCL3/PHF19* locus encodes two isoforms: a hPCL3L/PHF19L full-length protein (580 AA) and a short isoform, hPCL3S/PHF19S (207 AA) which contains only the TUDOR domain, PHD1-the first of two PHD- domains and a specific C-terminal region (AA 155-207) generated by a read-through and the use of an alternative intronic polyadenylation site between exons 5 and 6 [21, 22] (Figure 1A). This PHD1 domain, which is very divergent between the three human Polycomb-like proteins, could be associated with specific functions for each orthologue, such as the stabilization of P53 in the case of PHF1 [23]. Indeed, PHF1 through its PHD1 domain is the only human Polycomb-like protein capable of inducing cell quiescence by interacting with P53 to stabilize it independently of its TUDOR domain and thus of its binding at chromatin [24].

In a recent study, *hPCL3S* has been shown to be up-regulated in hepatocarcinoma tumors (HCC) and cell lines and promoting their growth and migration through activation of the β-catenin/IL-6 pathway [25]. Indeed, hPCL3S has been shown to stabilize β-catenin through direct interaction and inhibition of components of its degradation complex, thereby increasing the expression of the Wnt/β-catenin pathway target gene, *IL6* [25]. Unfortunately, however, the exact contribution of the TUDOR and PHD1 domains to these interactions has not been investigated. Thus, Polycomb-like proteins appear as essential co-factors to regulate the transcriptional activity of the PRC2.1 complexes but are also involved in fundamental chromatin-independent mechanisms, whose deregulation could participate in tumorigenesis.

In this study, we have quantified the expression levels of both *hPCL3* isoforms in primary prostate tumors as well as in the hormone-dependent LNCaP and hormone-independent DU145 and PC3 prostate cancer cell lines. RT-qPCR experiments on 25 prostate tumors revealed that *hPCL3S* is overexpressed in 75% of cases. In addition, *hPCL3S* is overexpressed in the DU145 and PC3 hormone-insensitive cell lines, but not in the hormone-sensitive LNCaP cell line. Wound-healing and proliferation assays showed that siRNA-induced decrease of *hPCL3S* impaired the proliferation and migration properties of DU145 and PC3 cells. Conversely, the stable transfection of hPCL3S into LNCaP increased these properties. Stable transfection of wild-type hPCL3S or a TUDOR domain mutant (W50A) unable to bind H3K36me3 resulted in increased proliferation and migration of LNCaP whereas a PHD1 mutant decreased their proliferation. Collectively, our results provide insights into a new mechanism whereby AR-independent prostate cancer cell lines acquire heightened ability to proliferate and migrate and highlight *hPCL3S* targeting as a new potential interventional strategy against castration resistant prostate cancers.

## RESULTS

### hPCL3S is up-regulated in human primary prostate tumors

**Figure 2:**
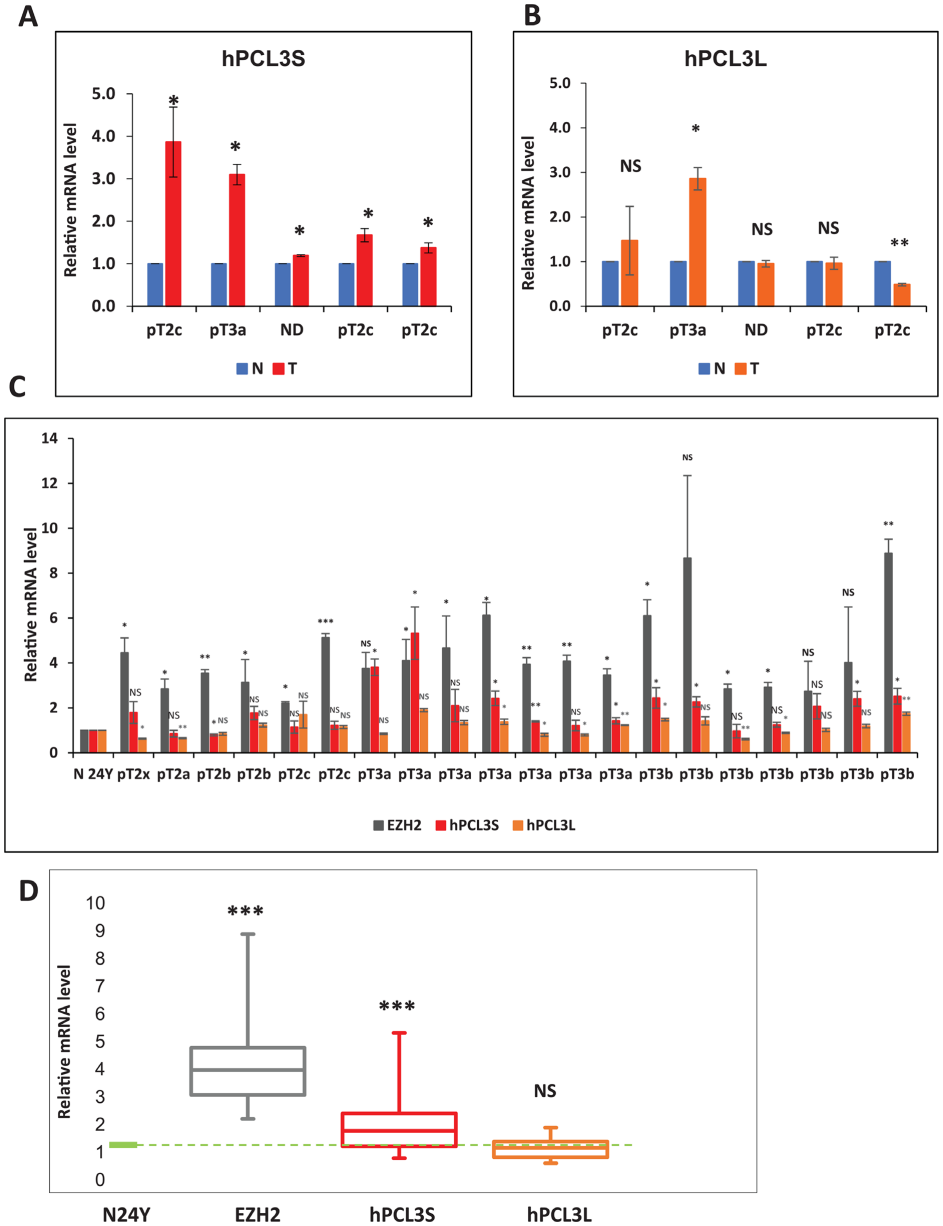
Analyses of *hPCL3S and hPCL3L* expression in human prostate cancer tissue. (**A–B**) Comparison of *hPCL3S* and *hPCL3L* mRNA levels in five matched prostate cancer (T) and normal adjacent (N) tissue samples by RT-qPCR analyses. (**C**) Expression in 20 tumor tissues of *hPCL3S*, *hPCL3L* and *EZH2*. The *hPCL3S*, *hPCL3L* and *EZH2* expression were measured by RT-qPCR analyses in comparison with their expression in a normal prostate tissue obtained from a young 24-years old donor (BioChain). (**D**) Quantification of EZH2, *hPCL3S* and *hPCL3L* expression in the 20 prostate tumors. The *hPCL3S*, *hPCL3L* and EZH2 expression measured in panel C is represented as box plots. The box area corresponds to the first and third quartile. The median is shown as a horizontal line in the box. The maximum and minimum of the values are indicated by the whiskers above and below the box.

Samples of human primary prostate cancers (PCas) were assessed for *hPCL3S and hPCL3L* expression using RT-qPCR analyses on total isolated RNAs (Supplementary Table 1). First, we determined the expression levels of *hPCL3S* and *hPCL3L* in 5 pairs of prostate cancer tissues and matched adjacent non-cancerous tissues (Figure 2A–2B and Supplementary Table 2). We found that in 3 cases out of 5, *hPCL3S* was expressed at higher levels than in the normal tissue (Figure 2A). However, the expression level of *hPCL3L* was not significantly higher in cancerous tissues than in normal tissue with the notable exception of one tumor with a pT3a TNM staging (Figure 2B).

In a second experiment, we analyzed a larger cohort of 20 prostate cancers including several aggressive tumors characterized by a high Gleason grading (from 7 to 9) and TNM staging (pT3a and pT3b) (Supplementary Table 1). Their *hPCL3S and hPCL3L* expression levels were determined using RT-qPCR analyses on total RNAs by comparison with total commercial RNAs prepared from the normal prostate of a young (24-years old) healthy donor (BioChain). The results showed that in 14 out of 20 samples the fold change of *hPCL3S* expression in the tumor tissue was above 1.5 relative to the normal prostate. Interestingly, the highest expression of *hPCL3S* was observed in some of the most advanced tumors, pT3a and pT3b according to the TNM classification for prostate cancer (Figure 2C and Supplementary Table 1). By contrast, the expression levels of *hPCL3L* remained highly homogenous (Figure 2C). As a further control, we analyzed the expression of EZH2. The data showed that EZH2 was up-regulated in all tumors (Figure 2C). All, these results are summarized using Tukey’s box-and-whisker plot (Figure 2D).

Thus, whereas *hPCL3L* expression levels do not show salient differences, *hPCL3S* is up-regulated in prostate tumors.

### hPCL3S is overexpressed in androgeno-independent prostate cell lines

*hPCL3S* is overexpressed in many cell lines [21, 22]. However, *hPCL3S* mRNAs levels in various types of transformed prostate cell lines have not been investigated in detail. Therefore, we measured the mRNA expression levels of *hPCL3S*, *hPCL3L* and *EZH2* in three transformed prostate cell lines; the androgeno-independent PC3 and DU145 and in the androgeno-dependent LNCaP in comparison with primary prostate epithelial cells PrEC and a non-tumorigenic prostate epithelial cell line, RWPE-1.

**Figure 3:**
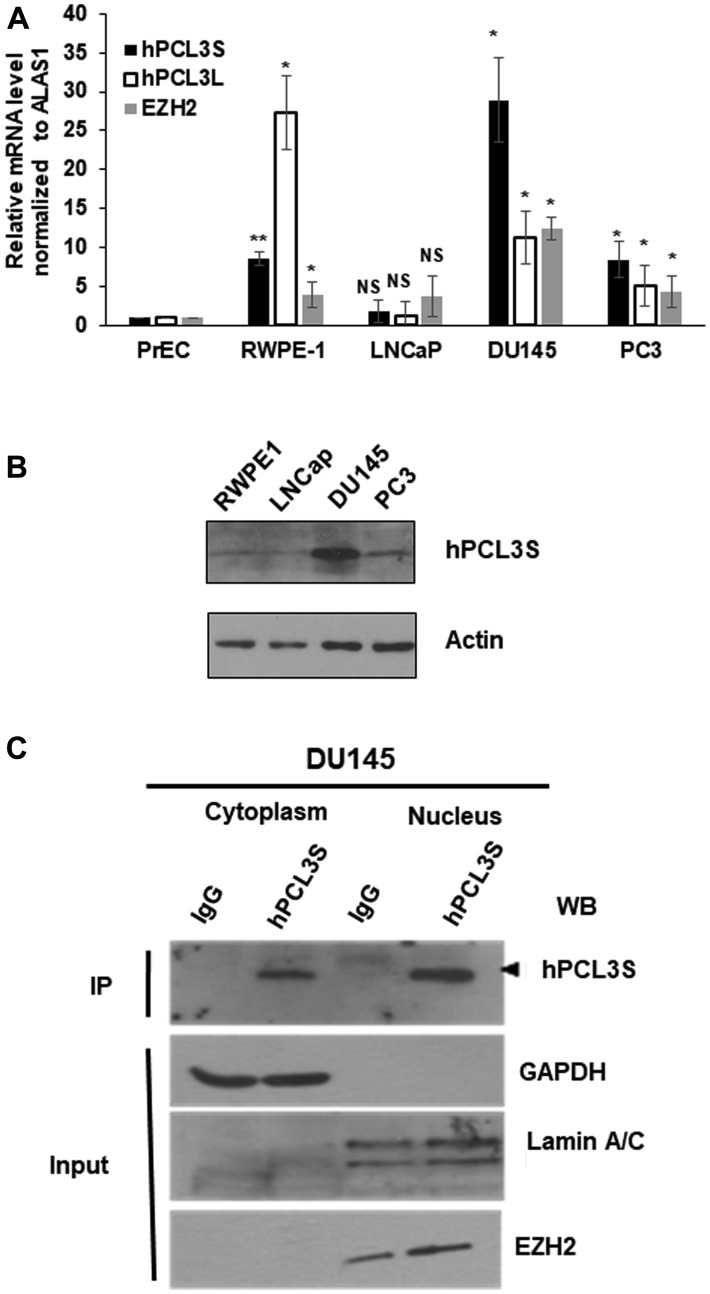
*hPCL3S* expression is specifically elevated in androgeno-negative prostate cancer cells, *in vitro*. (**A**) The expression levels of *hPCL3S, hPCL3L*, and *EZH2* were examined in normal primary (PrEC) and immortalized (RWPE-1) prostate epithelial cells as well as in transformed androgeno-dependent (LNCaP) and androgeno-independent (DU145 and PC3) prostate cancer cell lines by RT-qPCR analyses. (**B**) The expression levels of the hPCL3S protein in immortalized (RWPE-1) prostate epithelial cells and in transformed (LNCaP, DU145, and PC3) prostate cancer cell lines were examined using Western blotting. (**C**) Subcellular localization of endogenous *hPCL3S* proteins by cell fractionation experiments in DU145. Cytoplasmic and nuclear fractions prepared with the nuclear extraction Kit (Millipore) as previously described [22] were immunoprecipitated with rabbit IgG or anti-hPCL3S antibodies, resolved by SDS-PAGE and immunoblotted with anti-hPCL3S antibodies (top panel) to detect the endogenous hPCL3S proteins. To validate the accuracy of the cell fractionation, samples of each fraction were tested by Western blot with anti-GAPDH, anti-LAMIN A/C, and anti-EZH2 antibodies (bottom panels).

RT-qPCR analyses revealed that *hPCL3S* was strongly up-regulated in the two androgeno-independent cell lines and notably in the DU145 cell line as compared to PrEC whereas its expression level was very low in the androgeno-dependent LNCaP cell line. By contrast, *hPCL3L* was up-regulated in the immortalized RWPE-1 cells and in the two androgeno-independent cell lines, similarly to *EZH2* (Figure 3A). The expression levels of endogenous hPCL3S proteins in the various cell lines were analyzed by Western blotting (Figure 3B).

Previously, we demonstrated through transient transfection assays in HEK293T followed by immunofluorescence or cell fractionation experiments that FLAG-hPCL3L is almost exclusively found in the nuclear fraction [22], in accordance with its function as a Polycomb PRC2.1 cofactor [10]. By contrast, FLAG-hPCL3S was detected both in the nuclear and cytoplasmic fraction with a majority in the cytoplasm [22]. Cell fractionation experiments in DU145 cells confirmed that the endogenous hPCL3S proteins also displayed a mixed cytoplasmic and nuclear localization (Figure 3C).

In conclusion, *hPCL3S* is overexpressed in the androgeno-dependent DU145 and PC3 cell lines but not in the androgeno-independent LNCaP cell line which is characteristic of prostate adenocarcinomas. Therefore, we selected these cell lines to investigate the biological role of *hPCL3S* through siRNA-mediated knockdown and ectopic overexpression experiments.

### hPCL3S knockdown decreased cell growth and migration of the human androgeno-dependent prostate cancer cell lines, DU145 and PC3

**Figure 4:**
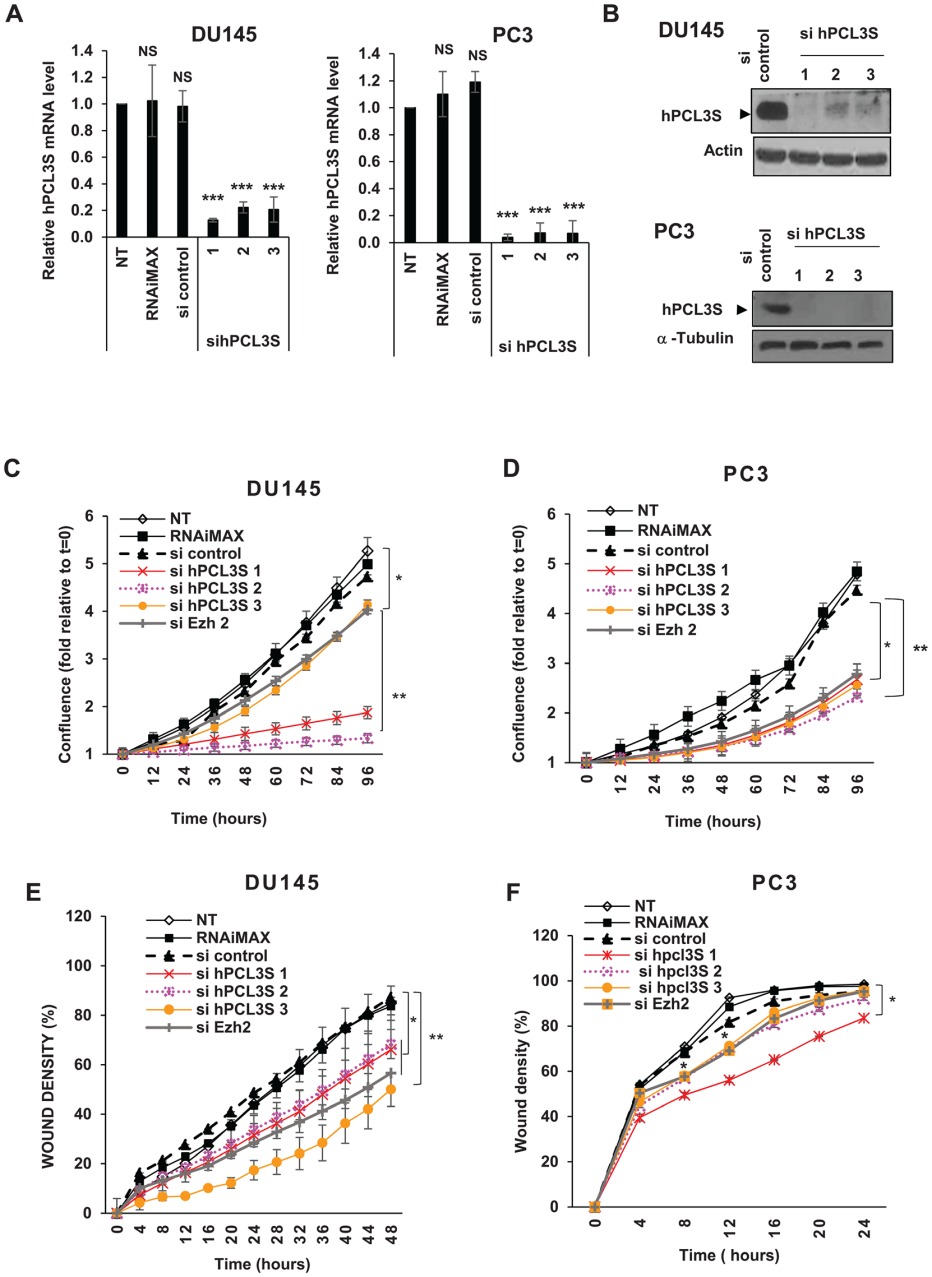
*hPCL3S* is essential for DU145 and PC3 cell proliferation and migration. (**A**) Validation of the *hPCL3S* siRNA efficiency by RT-qPCR analyses of transfected DU145 and PC3 cells. The expression level of *hPCL3S* was examined in the DU145 (left panel) and PC3 (right panel) prostate cancer cell lines transfected by the sicontrol RNA or each individual siRNA targeting *hPCL3S* by RT-qPCR analyses. As control, non-transfected LNCaP or LNCaP transfected by the transfection agent (RNAimax) alone were also tested. (**B**). Validation of the *hPCL3S* siRNA efficiency by Western blot analyses of transfected DU145 and PC3 cells. RNAs (used in, A) and proteins were simultaneously prepared from the same transfected cells. Total cell extracts were analyzed by Western blotting to confirm the knockdown of *hPCL3S*. Actin and α-tubulin were used as a loading control. (**C–D**) Knockdown of *hPCL3S* inhibited the cell proliferation of DU145 cells (C) and of PC3 cells (D). The proliferation of non-transfected cells and of cells transfected by RNAiMax alone or in combination with the indicated siRNA was examined using the Incucyte system. (**E–F**) Overexpression of *hPCL3S* inhibited the cell migration of DU145 cells (E) and of PC3 cells (E). The migration of non-transfected cells and of cells transfected by RNAiMax alone or in combination with the indicated siRNA was examined with the Incucyte Scratch wound system.

To explore the roles of *hPCL3S* in DU145 and PC3 cells, we silenced it through siRNA interference. Since available commercial *hPCL3*/*PFH19* siRNAs targeted both isoforms, we designed 3 siRNAs targeting the specific C-terminal part of *hPCL3S*. We verified by RT-qPCR and Western blot analyses that they efficiently inhibited *hPCL3S* expression (Figure 4A and 4B) with a relative lack of effect on *hPCL3L* (Supplementary Figure 1A).

Using these specific siRNAs, we investigated the effects of *hPCL3S* knockdown on the growth and migration of DU145 and PC3 cells using the Incucyte Live-Cell Imaging System. The cell proliferation curves as measured by the kinetics of cell confluence over a 96 hours time-course clearly indicated that *hPCL3S* knockdown dramatically decreased the proliferation of DU145 and PC3 cells using either the three individual siRNAs (Figure 4C and 4D) or a pool of these three siRNAs in DU145 to limit off-target effects (Supplementary Figure 1B). A similar cell growth inhibition effect is observed in DU145 and PC3 cells transfected by a siRNA targeting *EZH2*, the histone methyltransferase of the PRC2 complexes which is up-regulated in prostate cancers (Figure 4C and 4D).

Follow-up experiments showed that *hPCL3S* knockdown caused a severely impaired colony formation of DU145 cells in anchorage-independent growth conditions (Supplementary Figure 1C).

We, then, examined if *hPCL3S* knockdown could inhibit the migration properties of DU145 and PC3 cells. In a preliminary experiment, we performed wound-healing assays using DU145 cells. These experiments showed that, after 24 hours (a time window excluding the effects on cell growth upon *hPCL3S* loss), the wound closure was delayed in the absence of *hPCL3S* (Supplementary Figure 1D–1E). In order to accurately measure these effects, we used the Incucyte scratch wound system allowing us to follow cell migration in real time over a longer time period. Therefore, 24 h after siRNAs transfection, DU145 and PC3 cells were treated with low dose Mytomicin C (10 μg/ml) for 1 hour to inhibit their proliferation before wound scratch and monitoring of cell migration for 48 hours. These experiments clearly demonstrated that DU145 cell migration properties were severely impaired upon *hPCL3S* knockdown (Figure 4E). The effects of the hPCL3S siRNAs on PC3 migration was less clear probably because in that case the wound closure occurred very rapidly (Figure 4F). Taken together, these results suggest that *hPCL3S* plays an important role in the regulation of cell growth and migration of DU145 and PC3 cells that overexpress this variant.

### Transfection of LNCaP with p-AM-TAg expression vectors selected a PSA^-/lo^, AR^-^ and ALDH1A1^+^ cell population

**Figure 5:**
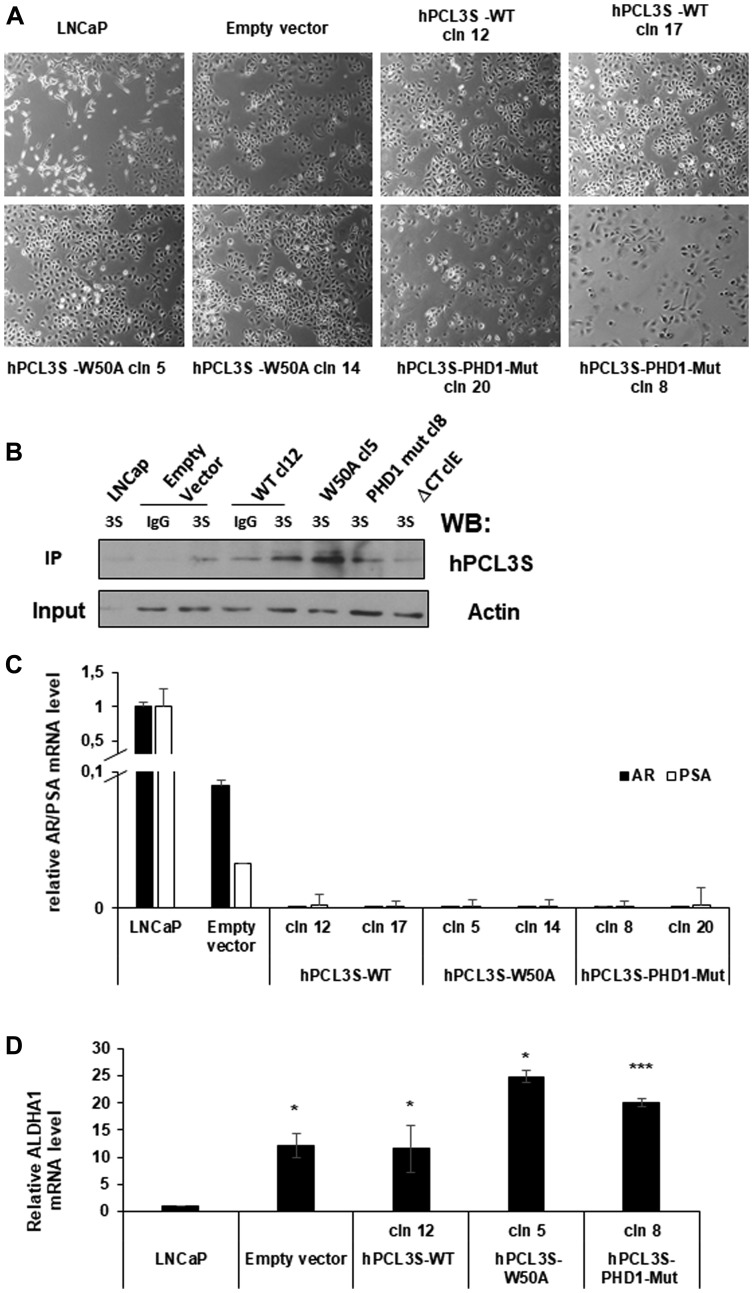
Selection of stable clones expressing the empty vector (p-AM) or the various versions of hPCL3S-AMTag fusion proteins by transfection selected a subpopulation of LNCaP cells. (**A**) Transfection of LNCaP with p-AMTag expression vectors selects a cell population with an epithelium-like morphology. Bright field images of parental LNCaP cells, of a pool of clones obtained after transfection of these LNCaP cells by the p-AM empty vector and of individual clones used in this study and obtained after transfection of hPCL3S wt (clones 12 and 17), hPCL3S W50A (clones 5 and 14) and hPCL3S Mut-PHD1 (clones 8 and 20). (**B**) Imunoblotting analyses of lysates prepared from LNCaP or LNCaP clones obtained after transfection with the various p-AM-Tag expression vectors. Immunoprecipitation analyses were performed with normal rabbit Immunoglobulins (IgG) or with commercial rabbit antibodies generated against anti GST-PCL3S (3S) and followed by Western blot analyses with commercial goat antibodies generated against a C-terminal-peptide (hPCL3S). Please note that this experimental strategy did not allow visualizing the Delta-C-term E mutant. A faint non-specific band is detected in the wtCl2 IgG lane and in the hPCL3S (3S) lanes for empty vector and Delta C-term E. 2% of each total lysates before immunoprecipitation was kept as Input and analyzed by Western blotting with actin antibodies as loading control. (**C**) RT-qPCR analyses of *AR* and *PSA* expression level in the parental LNCaP cells, in LNCaP transfected by the empty vector as well as in the various hPCL3S (wt and mutants) stable clones obtained. (**D**) Similar RT-qPCR analyses for *ALDH1A1* expression level in the indicated cells.

Overexpression of *hPCL3S* in LNCaP was achieved using the p-AM-Tag expression vector which appends the AM-tag sequences to the C-terminal part of the protein and allows puromycine selection of stable clones either with increased expression of *hPCL3S* or transfected with the empty vector as control (Figure 1B). Strikingly, we noticed that whereas our parental LNCaP cultures displayed two types of cells with clearly distinct morphologies, all the clones emerging from our transfection with Lipofectamine followed by puromycine selection were highly homogenous with a population of small highly refringent epithelial-like cells (Figure 5A). This holds true for cells transfected with the empty vector (CaP-pAM) as well as cells transfected with wt *hPCL3S* or with the majority of the different *hPCL3S* mutants analyzed in this study. The expression levels of the various hPCL3S-AM-Tag proteins in the stable clones generated in this study (see below) were roughly similar as show by immunoprecipitation followed by Western blotting (Figure 5B).

LNCaP cultures are known to be highly heterogeneous and to contain cells with high levels of AR and PSA together with cells expressing low levels of both AR and PSA. These PSA^-/lo^ cells have a high clonogenic capacity [26, 27]. RT-qPCR analyses demonstrated that the high expression levels of PSA and AR in our bulk LNCaP cultures are dramatically reduced in the controls transfected with the empty vector and virtually undetectable in the clones expressing the various versions of *hPCL3S* (Figure 5C). This PSA^-/lo^ population is characterized by high Aldehyde Dehydrogenase activity [26]. RT-qPCR analyses demonstrated that in contrast with the parental LNCaP cells all the selected clones obtained either with the empty vector or with the various hPCL3S variants used in these studies all expressed *ALDH1A1*, which is considered as a stem cell marker [28] (Figure 5D).

In conclusion, the protocol (Lipofectamine transfection followed by Puromycine selection) that we have used to obtain stable clones, mainly sustained the growth of PSA^-/lo^, AR^-^, and ALDH1A1^+^ cells.

### Overexpression of hPCL3S increases proliferation and migration of LNCaP cells

**Figure 6:**
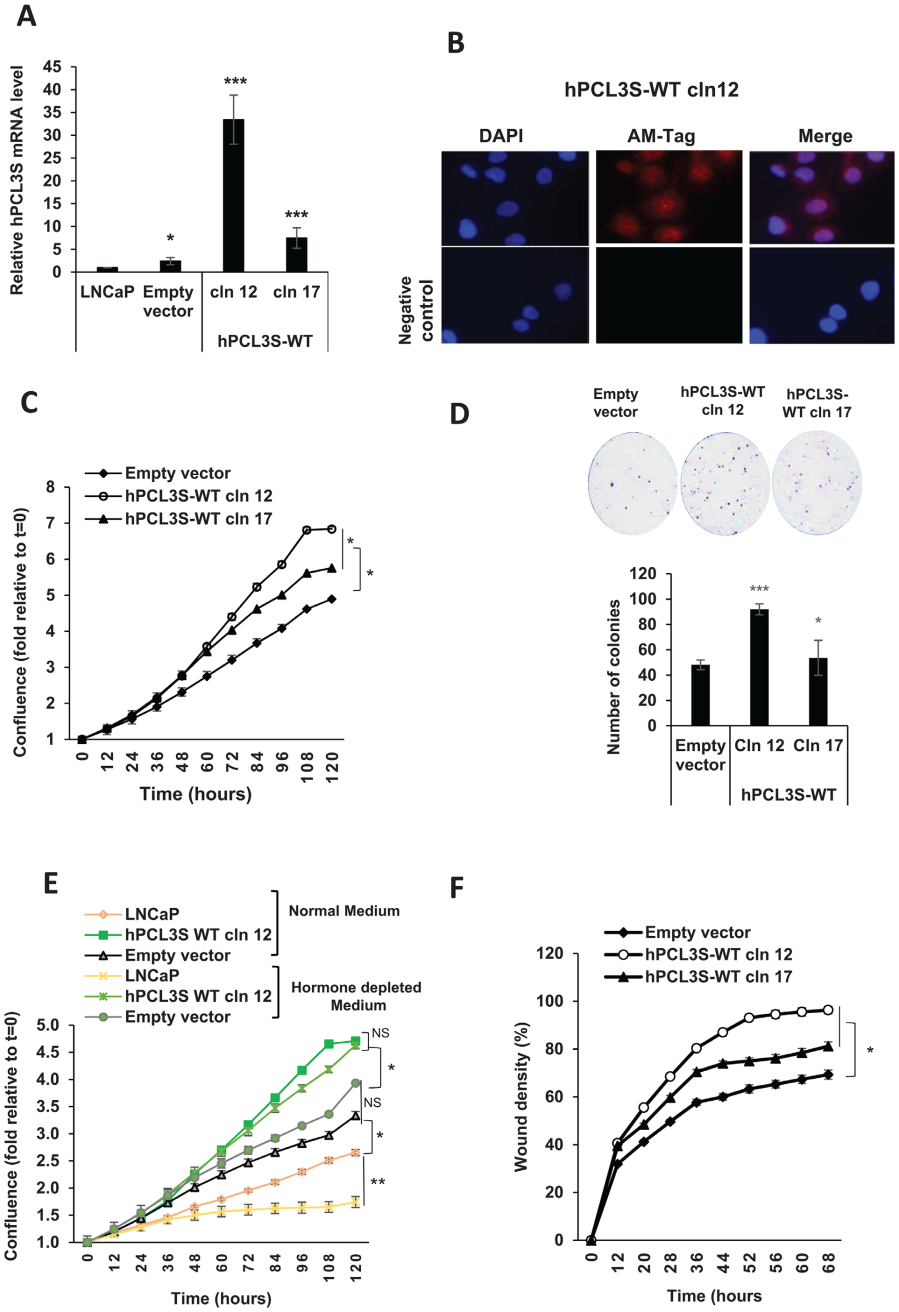
*hPCL3S* promotes proliferation and migration in the androgeno-dependent human prostate cancer cells, LNCaP. (**A**) Stable overexpression of hPCL3S in LNCaP prostate cancer cells. Quantitative PCR analyses of *hPCL3S* expression was performed on LNCaP cells stably transfected with the empty vector (pool of clones) or on individual clones obtained after transfection of the hPCL3S-AMTag expression vector. (**B**) Immunofluorescence analyses of LNCaP-hPCL3S-clone 12. The three top panels correspond to the DAPI staining, the conventional immunofluorescence analysis with the anti-AMTag antibody and the merging of the two images, respectively. The bottom panel represents the same experiment except that the primary anti-AMTag antibody was omitted (negative control). (**C**) Overexpression of *hPCL3S* promoted the cell proliferation of LNCaP cells. The proliferation of parental LNCaP cells as well of a pool of empty vector transfected clones or the hPCL3S overexpressing clones 12 and 17 was examined using the Incucyte system. (**D**) Clonogenicity assays. The empty vector transfected cells and the hPCL3S overexpressing clones 12 and 17 were compared in a clonogenicity assay. An example of the crystal blue staining (1 picture out of three) is shown as well as a graphical view of the three samples for each condition (see Supplementary Figure 3 for details). (**E**) Overexpression of *hPCL3S* promoted the cell proliferation of LNCaP cells in hormone-depleted medium. The proliferation of parental LNCaP cells as well of a pool of empty vector transfected clones or the hPCL3S overexpressing clones 12 grown in normal medium (RPMI 1640 +10% fetal calf serum) or in hormone-depleted medium (RPMI 1640 +10% charcoal-stripped fetal calf serum) was examined using the Incucyte system. (**F**) Overexpression of *hPCL3S* promoted the cell migration of LNCaP cells. The migration of parental LNCaP cells as well of the various stable clones was examined using the Incucyte Scratch Wound system.

We, next, wanted to determine more precisely the effects due to the overexpression of wt hPCL3S and of its various mutants. Then, as a first step, we studied some randomly picked individual clones overexpressing wt hPCL3S-AMTag. RT-qPCR analyses after clone selection indicated that LNCaP-hPCL3S-cln (clone) 12 and -cln 17 had approximately 30 and 8 fold more *hPCL3S* respectively relative to LNCaP or to a pool of empty vector-transfected LNCaP cells (Figure 6A). Immunofluorescence analyses of hPCL3S-cln 12 detected the hPCL3S-AMTag fusion protein which also displayed a mixed nuclear and cytoplasmic localization (Figure 6B), as shown by cell fractionation experiments for the endogenous hPCL3S proteins in DU145 (Figure 3B) [22].

Then, the proliferation and migration properties of these clones were analyzed using the Incucyte Live-Cell Imaging System. First, the proliferation curves demonstrated that *hPCL3S* overexpression in two independent clones increased the proliferation potential of LNCaP cells as compared to the empty vector control (Figure 6C). These results were independently confirmed using the anchorage-independent growth assay (Figure 6D).

In agreement with their hormone-dependency, LNCaP cells proliferate less efficiently in hormone-depleted medium LNCap cells (Figure 6E). By contrast, the proliferation curves of LNCaP transfected with the empty vector or with the hPCL3S expression vector which have lost expression of AR were highly similar in normal and in depleted medium (Figure 6E).

Similarly, the results obtained in wound healing assays (Supplementary Figure 2) or in the migration assay with the Incucyte Scratch wound system clearly showed an increase in migration for the *hPCL3S* overexpressing clone 12 (Figure 6F).

Thus, ectopic overexpression of *hPCL3S* in LNCaP cells increased their proliferation, anchorage-independent and migration properties. Interestingly, these phenotypic changes were correlated with hPCL3S levels in these subclonal populations (Figure 6, compare clone 12 high-hPCL3S- and clone 17 low-hPCL3S).

### The hPCL3S specific C-terminal end generated by intronic alternative polyadenylation (CR-APA) did not significantly contribute to the increase of proliferation and migration

**Figure 7:**
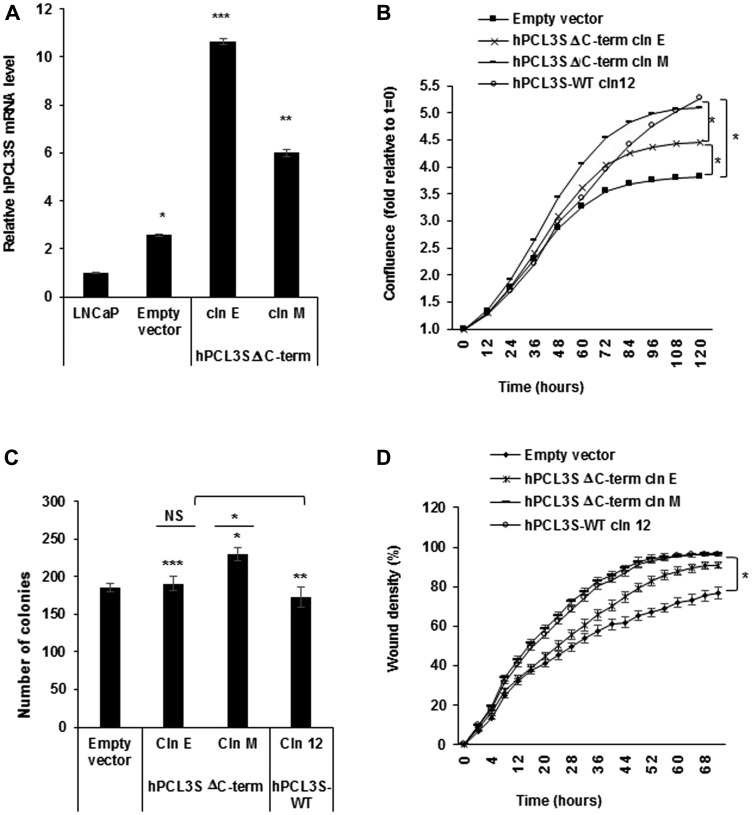
The short (AA 155-207) specific C-terminal end of *hPCL3S* generated by the alternative polyadenylation mechanism is not essential for the promotion of proliferation and migration. (**A**) Stable overexpression of hPCL3S-Delta C-term in two individual clones was analyzed by RT-qPCR analyses. (**B**) Overexpression of *hPCL3S-ΔC-Term* promoted the proliferation of LNCaP cells as efficiently as wt *PCL3S.* The proliferation of LNCaP transfected by the empty vector, of *hPCL3S* overexpressing clone12 and of two clones overexpressing the Delta C-term mutant was examined using the Incucyte system. (**C**) The effect of the overexpression of hPCL3S-ΔC-Term was examined using the clonogenicity assay (the original pictures used for this graphical view are shown in Supplementary Figure 3). (**D**) Overexpression of *hPCL3S-ΔC-Term* promoted the cell migration of LNCaP cells as efficiently as *wt hPCL3S.* The migration of the various indicated clones was examined using the Incucyte Scratch Wound system.

*hPCL3S* is generated through the usage of an internal alternative intronic polyadenylation site [21]. This mechanism known as CR-APA (Coding Region Alternative PolyAdenylation) produces different protein isoforms endowed with different properties [29, 30]. Indeed, the C-terminal moiety of hPCL3L which contains several important and well characterized functional domains (the PHD2, the “Winged-helix” (EH) and the “RC” SUZ-12 binding domain) (Figure 1A) is lost in hPCL3S where it is replaced by a short (AA 155-207) specific C-terminal end of unknown function [21]. However, analyses of two stable clones of LNCaP cells transfected with a C-terminal deletion mutant (Δ155-207), hPCL3S-ΔC-term, failed to demonstrate any global salient differences as compared to the wild-type *hPCL3S* isoform in terms of proliferation, clonogenicity and cell migration properties (Figure 7A–7D).

Thus, the specific C-terminal end created by the CR-APA mechanism is not clearly involved in the regulation of proliferation and migration by *hPCL3S*.

### Proliferation and migration effects are independent of the H3K36me3 binding activity of the hPCL3 TUDOR domain

The TUDOR domain responsible for the binding to H3K36me3 is the major functional domain found both in the full-length hPCL3L protein and in the shorter hPCL3S isoform.

The residue W50 in the TUDOR domain is essential for the binding of PCL3/PFH19 to H3K36me3 and the genome-wide deposition of H3K27me3 by PRC2 [16, 17, 18, 31].

**Figure 8:**
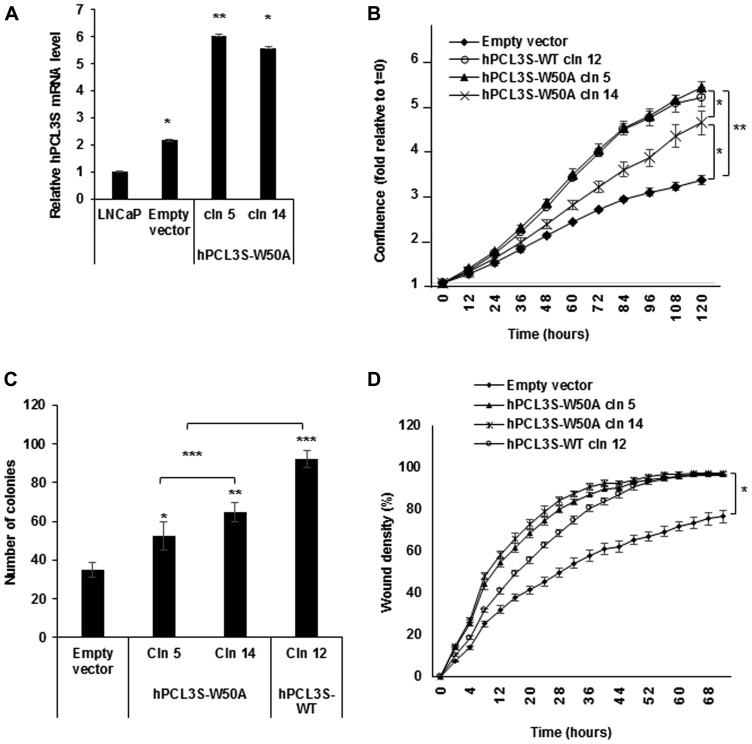
The H3K36me3 binding activity of the *hPCL3S* TUDOR domain is not required for the promotion of proliferation and migration. (**A**) Stable overexpression of hPCL3S-W50A in two individual clones was confirmed by RT-qPCR analyses. (**B**) The W50A point mutation in the TUDOR domain did not impaired the proliferation of LNCaP cells as compared with *wt hPCL3S.* The proliferation of empty vector transfected cells or wt *hPCL3S* overexpressing clone12 was compared to two different clones overexpressing *hPCL3S-W50A* (clones 5 and 14) using the Incucyte system. (**C**) The effect of the overexpression of hPCL3S W50A was examined using the clonogenicity assay (the original pictures used for this graphical view are shown in Supplementary Figure 4). (**D**) Overexpression of *hPCL3S W50A* promoted the cell migration of LNCap cells as efficiently as *wt PCL3S.* The migration of the various stable clones was examined using the Incucyte Scratch Wound system.

We therefore generated the hPCL3S W50A point mutant construct selectively inhibiting this essential function of the TUDOR domain, obtained stable transfected clones in LNCaP cells and analyzed their properties with the Incucyte system, as described above. Strikingly, this mutant predicted to be unable to bind to H3K36me3 maintained proliferation, anchorage-independent growth and migration properties similar to hPCL3S wt on LNCaP cells (Figure 8).

As a whole, these results strongly suggested that the proliferative and migration effects mediated by *hPCL3S* overexpression are not due to the perturbation of H3K36me3 binding by hPCL3L through a dominant-negative mechanism resulting in a genome-wide deregulation of H3K27 methylation by Polycomb PRC2.1 complexes.

### The hPCL3 PHD1 is implicated in the increase of proliferation and migration

**Figure 9:**
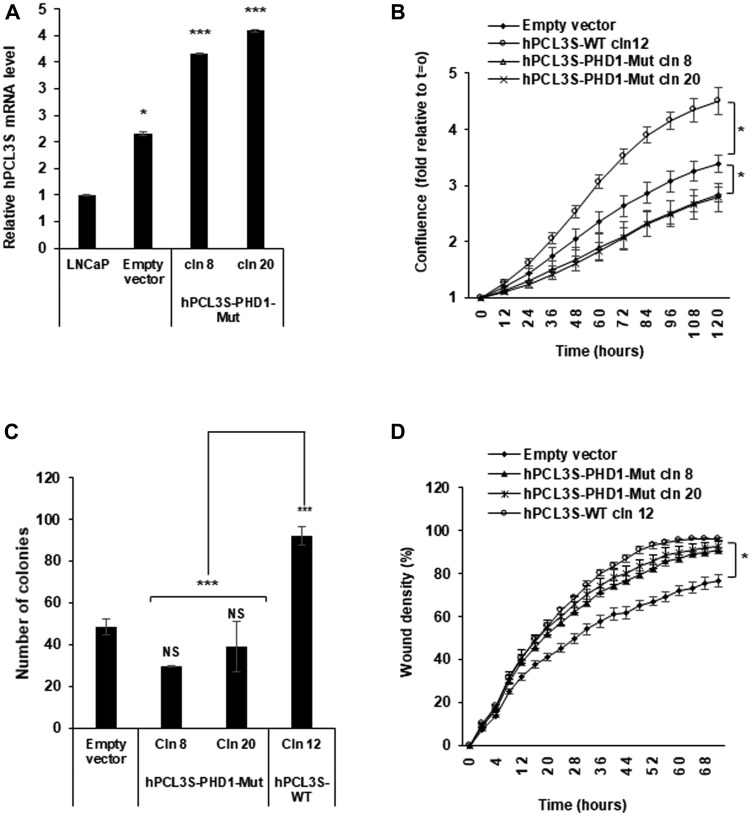
The PHD1 domain is essential for the promotion of proliferation. (**A**) Stable overexpression of hPCL3S-PHD1-Mut in two individual clones was confirmed by RT-qPCR analyses. (**B**) The mutation of an essential β strand in the PHD1 domain impaired the proliferation of LNCaP cells as compared with *wt hPCL3S.* The proliferation of empty vector transfected cells or wt *hPCL3S* overexpressing clone12 was compared to two different clones overexpressing hPCL3S-PHD1-Mut (clones 8 and 20) using the Incucyte system. (**C**) The effect of the overexpression of hPCL3S-PHD1-Mut was examined using the clonogenicity assay (the original pictures used for this graphical view are shown in Supplementary Figure 4). Error bars correspond to standard deviation between the empty vector and each clone. The ^***^ above the brackets corresponds to the comparison of the wt clone 12 with the two PHD1-mutated clones. (**D**) Overexpression of hPCL3S-PHD1-Mut slightly promoted the cell migration of LNCaP cells as compared to the control empty vector but less efficiently than *wt PCL3S.* The migration of the various stable clones was examined using the Incucyte Scratch Wound system.

The other functional domain conserved in hPCL3S is the first of the two PHD (Plant Homeo Domain), PHD1 (Figure 1A). In contrast with the PHD2 domain which is well conserved and involved in interaction with PRC2 components [22], PHD1 is not conserved and seems to be implicated in ortholog-specific interactions, as shown for the interaction of PHF1 with P53 [23]. To address the function of the hPCL3S PHD1 domain, we generated a mutant construct targeting Cysteine 3 in the PHD structure as well as an adjacent highly conserved region of hydrophobicity identified by sequence alignments of various PHD domains [32, 33] (Figure 1B). This mutant (E^112^ILIC^116^ to AQQQA) is located in an essential β strand involved in the interaction of PHD domains with several partners. Indeed, a similar QQQA mutant abolished the interaction between Drosophila PCL and EZH2 [34]. It contains also a residue (I^115^ in PCL3/PHF19 and PCL2 replaced specifically by a Serine in PHF1) essential for the interaction between PHF1 and P53 [23]. Finally, a point mutation of the conserved acidic residue (equivalent to hPCL3S E^112^) in the PHD domain of CBP abolishing its Histone acetyl transferase activity has been identified in the Rubinstein-Taybi syndrome [35]. Two stably transfected clones of LNCaP cells expressing this PHD1-Mut-hPCL3S construct proliferated significantly at a lower rate that LNCaP cells overexpressing hPCL3S (cln12), as shown by Incucyte analyses (Figure 9A and 9B). In clonogenicity assays, these two PHD1-Mut-hPCL3S constructs gave rise to a lower number of colonies than the pool of empty vector transfected cells (Figure 9C). Interestingly, these PHD1 mutations did not significantly impair the migration potential of stably transfected LNCaP cells (Figure 9D).

Thus, the hPCL3S PHD1 domain seems involved in the proliferative effects but not in the cell migration increases induced by *hPCL3S* overexpression.

### hPCL3S did not significantly regulate the Wnt/β-catenin pathway in prostate cancer cell lines

In a recent study, *hPCL3S* has been shown to be up-regulated in hepatocarcinoma tumors (HCC) and cell lines and to promote their growth and migration through activation of the β-catenin/IL-6 pathway [25]. Indeed, hPCL3S has been proposed to stabilize β-catenin through direct interaction and inhibition of components of its degradation complex, thereby increasing the expression of the Wnt/β-catenin pathway target gene, *IL6* [25].

**Figure 10:**
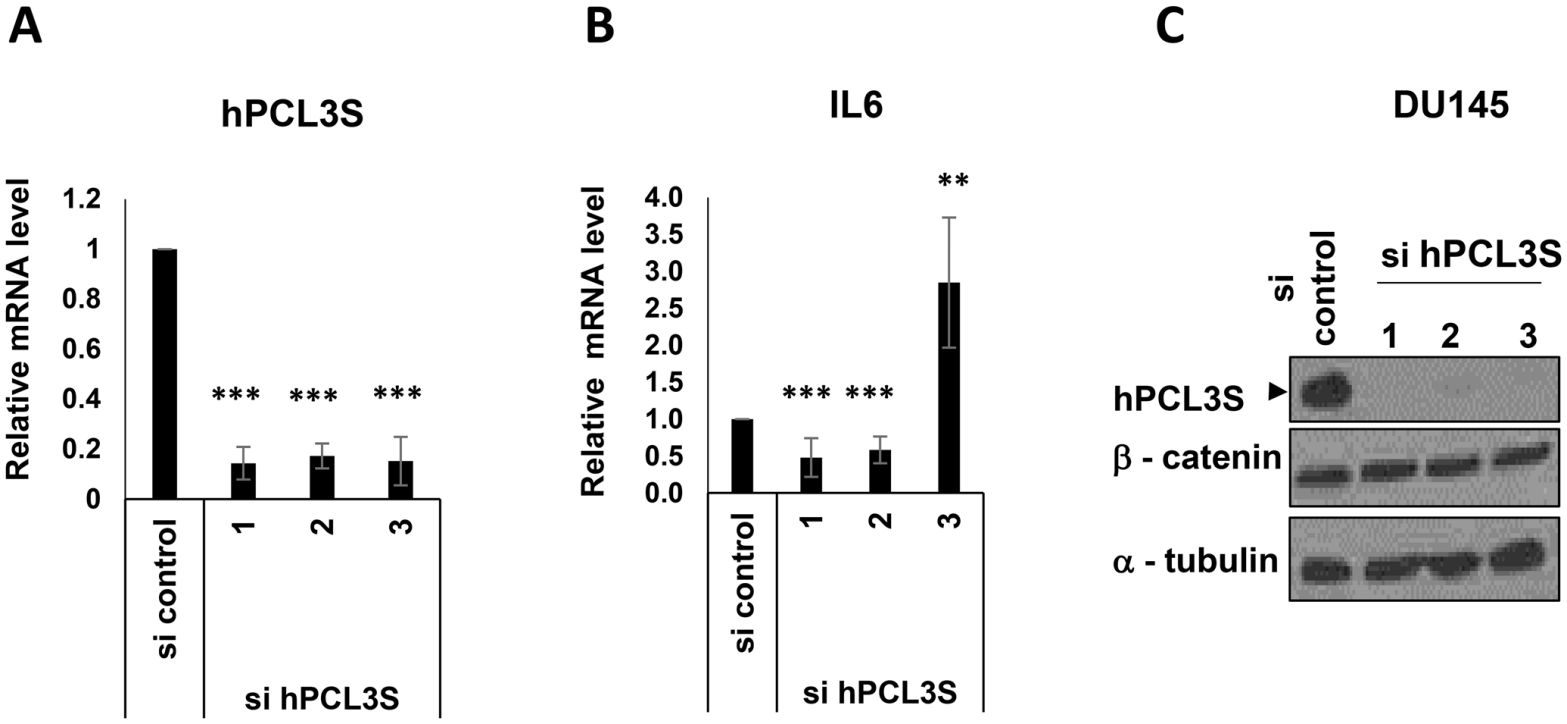
IL6 appeared as a hPCL3S target gene but independently of βcatenin stabilization in DU145 cells. After transfection of DU145 cells by *hPCL3S* siRNAs, RNAs and proteins extracts were simultaneously prepared from the same cells. (**A**) Efficient knockdown of *hPCL3S* in DU145 after transfection of the three individual siRNAs was confirmed by RT-qPCR analyses. (**B**) *IL6* expression is down regulated after transfection of two out of three individual hPCL3S siRNAs. The RT samples analyzed in panel A were checked for expression of *IL6* by qPCR. (**C**) Efficient knockdown of *hPCL3S* did not affect the βcatenin protein levels. Total proteins extracts were tested by Western blot for the expression of hPCL3S, βcatenin, and Tubulin as a loading control.

Using our three siRNAs efficiently targeting *hPCL3S*, we did not observe a consistent impact on *IL6* expression in DU145 cells (Figure 10A and 10B). We next examined the β-catenin protein levels by Western blot analyses of the same cell lysates. Strikingly, we failed to detect any significant decrease of β-catenin expression after knockdown of *hPCL3S* in these cells (Figure 10C). Furthermore, we were unable to detect a significant interaction between hPCL3S and the E3 ligase for β-catenin, β-TrCP using transient transfection assays in HEK293T cells (Supplementary Figure 5).

Thus, whereas *hPCL3S* seemed to be somehow implicated in the regulation of *IL6* expression, the mechanism is independent of the activation of the Wnt/β-catenin pathway in prostate cancer cells and might thus involve another cell-specific signaling pathway.

Thus, our knock-down experiment suggested that *hPCL3S* seemed to be somehow implicated in the regulation of *IL6* expression. However, the mechanism in prostate cancer cells is independent of the activation of the Wnt/β-catenin pathway, as previously demonstrated for hepatocarcinoma cell lines and might thus involve another cell-specific signaling pathway.

### Effects of hPCL3S overexpression in LNCaP and validation of differentially expressed genes

**Figure 11:**
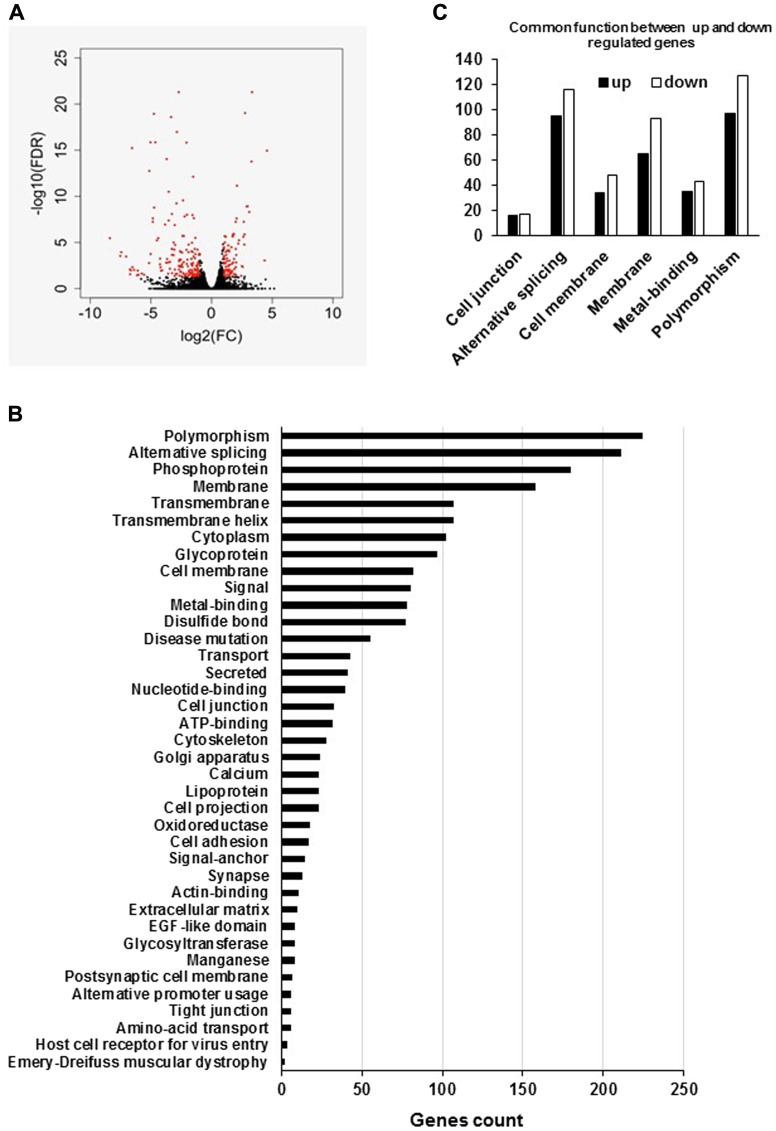
Global analyses of the RNA-Seq data of LNCaP empty vector and LNCaP cells overexpressing hPCL3S (Clone 12). (**A**) Volcano plot shows gene expression changes upon PHF19S induction in LNCap cells. X-axis shows fold changes in expression and y-axis shows adjusted *p* values. Red dots indicate the genes differentially expressed (at least two-fold changes and *p*-value < 0.05). (**B**) The enriched pathways were identified using the Database for Annotation, Visualization and Integrated Discovery (DAVID). (**C**) Graphical representation of the common functions between up- and down-regulated genes.

In an attempt to decipher the molecular mechanisms by which *hPCL3S* overexpression promotes cell growth and proliferation, we performed RNA-Seq analyses in a pool of stable clones obtained after transfection of LNCaP cells by the empty p-AM vector used as controls and in a clone of LNCaP overexpressing *hPCL3S*, LNCaP-hPCL3S cln12 cells (Figure 11A). We then defined a list of 240 statistically significant differentially expressed genes in the LNCaP clone 12 overexpressing hPCL3S as compared to control (empty vector) cells (1.5 fold changes and adjusted *p* value < 0.05). Among them, 84 genes were up-regulated and 156 genes down regulated (Supplementary Table 3). Functional enrichment analyses on the global RNA-Seq data performed using the DAVID (Database for Annotation, Visualization and Integrated Discovery) database identified Polymorphism, Alternative splicing, Phosphoprotein and Membrane as the four more enriched pathways with 224, 211, 180 and 158 genes identified respectively (Figure 11B and 11C).

**Figure 12:**
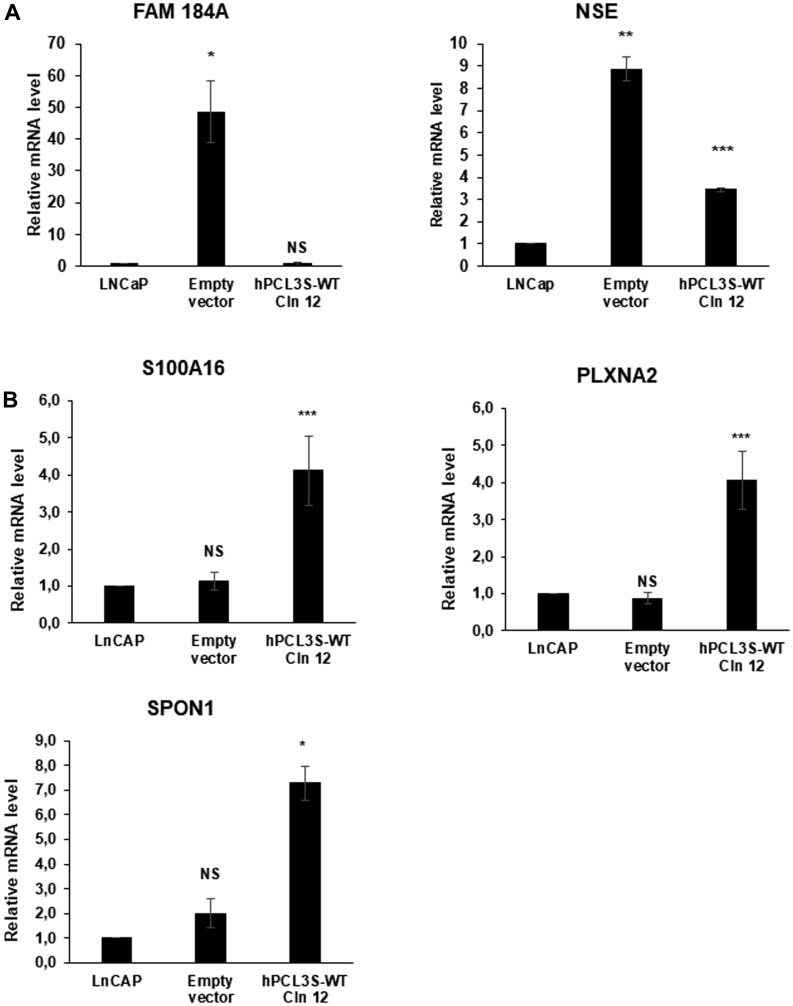
Validation of some differentially expressed genes. (**A**) *FAM1484A* is up-regulated in LNCaP transfected by the p-AM empty vector but down-regulated in LNCaP clone 12 cells surexpressing *hPCL3S*. RNAs were extracted from the non-transfected LNCaP cells as well as from cells transfected with the empty vector or with the p-AM-hPCL3S wt clone 12. The expression of Neuron-specific enolase, a classical marker of NED, has also been tested. (**B**) Validation by RT-qPCR analyses of the RNA-Seq data showing differential regulation of some selected genes in the LNCaP clone 12 cells. Selected genes (*S100A16*, *SPON1* and *PLXNA2*) were analyzed by RT-qPCR and showed the expected differential regulation.

The most down-regulated gene was *FAM184A* (family with sequence similarity 184 member A), a gene broadly expressed among various human tissues including prostate but with no known function. Interestingly, *FAM184A* has been found in a list of 88 pan-NET genes which are up-regulated in Neuroendocrine tumors (NETs) versus non-NETs in three different cancer types; prostate, lung and nervous system cancer [36]. Neuroendocrine tumors are a highly aggressive variant of prostate cancer often emerging during progression of the disease, notably at the castration resistant stage [2]. Validation by RT-qPCR analyses demonstrated that whereas it is not expressed in the parental LNCaP bulk cell population, *FAM184A* is highly up-regulated in the clones obtained after transfection with the empty expression vector (Figure 12A). A similar “spontaneous” neuroendocrine transdifferentiation of LNCaP has been described in a study addressing the role of the Wnt activator FOXB2 after stable transfection in LNCaP of a control empty pcDNA3 vector followed by G418 selection [5]. In close agreement with the RNA-Seq data, overexpression of *hPCL3S* in LNCaP clone 12 strongly repressed the expression of *FAM184A* and even to a very low level as compared to non-transfected LNCaP cells (Figure 12A). Similarly, when we analyzed the expression of neuron-specific enolase (NSE), a classical marker of neuroendocrine tumors, the strong up-regulation observed in empty vector transfected cells as compared to LNCaP (10× fold) is reduced to a 4× fold up-regulation in cells overexpressing hPCL3S (Figure 12A). These results further confirmed the major differences between the bulk LNCaP cells and the cell populations obtained after transfection and selection (Figure 5). In addition, the neuroendocrine transdifferentiation process induced in LNCaP transfected with the p-AM empty vector is counterbalanced by the overexpression of *hPCL3S*.

We next selected genes which have been previously implicated in prostate cancers and analyzed their expression by RT-qPCR analyses. Among them, we focused on three genes which appeared to be up-regulated in the RNA-Seq analyses: *S100A16*, a member of the S100 calcium-binding protein family up-regulated in many cancers [37] and promoting cell migration and metastasis in prostate cancer [38]; *Spondin-1* (SPON1), a protein of the extracellular matrix which is up-regulated in an orthotopic model in mice of PC3-derived tumors that frequently metastasized [39] and *PlexinA2* (PLXNA2), a semaphorin receptor up-regulated by the TRMPSS2-ERG fusion in metastatic cancers [40]. RT-qPCR analyses validated them as potential target genes up-regulated by ectopic expression of *hPCL3S* in LNCaP clone 12 cells (Figure 12B).

Collectively, our results strongly suggest that hPCL3S could promote proliferation and migration of prostate cancer cells through the up-regulation of previously characterized genes such as *S100A*16, *PlexinA2* or *Spondin1* which are known to be important for the proliferation and/or migration of prostate cancer cells.

## DISCUSSION

Prostate cancer is a complex multifactorial disease caused by a series of genetic and epigenetic alterations. Despite significant recent progress, prostate cancer remains a leading cause of cancer-related death due to the inescapable occurrence of resistance during conventional androgen deprivation therapy (ADT), mostly through neuroendocrine transdifferentiation, ultimately resulting in metastatic castration resistant tumors. These mechanisms are still poorly understood and hence an intense area of study.

In this study, we identified *hPCL3S* as a new candidate overexpressed in primary human prostate tumors and interestingly in hormono-independent (DU145 and PC3) but not in hormone-dependent (LNCap) cell lines. Our results suggest that *hPCL3S* over expression can endow cells with two properties essential for prostate cancer progression, namely increased growth and increased mobility. These effects are independent of the well-conserved H3K36me3-binding TUDOR domain but rather rely on the PHD1 domain which displays great variation among the three human Polycomb-like homologs [23].

In their seminal paper characterizing the human *PCL3/PHF19* locus, Wang et al. identified the full-length protein hPCL3L highly homologous to the PHF1 and PCL2 orthologues as well as a specific shorter isoform called hPCL3S [21]. This isoform is generated through alternative polyadenylation (APA), a widespread and major mechanism allowing a single gene to encode multiple RNA transcripts and affecting gene expression qualitatively and/or quantitatively. Indeed, when the alternative polyadenylation site is located in internal introns/exons, this mechanism known as CR-APA (coding-region-APA) can give rise to different protein isoforms with different C-terminal ends whereas the same protein is produced but from differentially regulated mRNAs if the alternative polyadenylation site is found in the 3’untranslated region (UR-APA) [29, 41]. These APA mechanisms can contribute to several human diseases and cancer, as recently shown by the widespread inactivation of tumor suppressor genes through CR-APA in leukemia [42].

This CR-APA mechanism produces different protein isoforms endowed with different properties such as the membrane-bound or secreted forms of Immunoglobulin M heavy chain or the cancer-specific Cyclin D1b isoform which is constitutively nuclear and devoid of regulatory motifs as well as of an essential regulatory phosphorylation site found in the C-terminus of Cyclin D1 [43]. In hPCL3S, the C-terminal three-quarters of hPCL3L (AA 155-580) are replaced by a short specific C-terminal end (AA 155-207) (Figure 1A) which is found conserved by BLAST analyses only in 6, mostly, primate species (Supplementary Figure 6) and which appears not to be required for the acquired growth and mobility increases upon hPCL3S overexpression (Figure 7). Thus, the major functional difference between hPCL3S and hPCL3L is the loss of several domains involved in the interaction with core PRC2 components to generate the PRC2.1 subtype, namely the PHD2 domain and the Reverse Chromodomain [15, 22] as well as in its recruitment to chromatin through the “Winged-Helix” EH domain [19, 20] while keeping the well characterized H3K36me3-binding TUDOR domain [16, 17, 18] (Figure 1). Therefore, one plausible hypothesis about the effects of hPCL3S overexpression on LNCaP cells was that this CR-APA mechanism generated a heavily truncated protein with a dominant-negative activity on the genome-wide deposition of H3K27me3 by PRC2.1 repressive complexes. However, our results strongly argue against this model. We have introduced in hPCL3S a W50A TUDOR point mutation which in the context of the full-length PFH19/hPCL3L protein inhibits H3K27me3 deposition in ES cells knocked-down for wild-type PHF19 [16, 17, 18]. Nevertheless, ectopic expression of this W50A hPCL3S mutant in LNCaP cells was able to increase their growth and mobility as efficiently as wild-type hPCL3S (Figure 8).

Our results rather sustain an alternative hypothesis which highlights the important role played by the PHD1 domain since a PHD1-mutated construct has lost its properties to increase proliferation and anchorage-independent growth but strikingly not to increase migration. The PHD1 domain is highly divergent between the three human PCL orthologs and even in the phylogeny of PCL3 proteins, strongly suggesting that this domain could be involved in ortholog-specific function. Our mutated construct target a β-strand involved in many aspects of PHD domain function including interaction of PCL with EZH2 [34] and stabilization of P53 by PHF1 [23]. However, the PHD domain is a protein-protein interaction domain as well as an epigenetic reader of histone marks predicted to adopt a cross-brace topology with 8 cysteine coordinating 2 zinc atoms and creating two interacting loops [33, 44]. This structure suggests that PHD domains could interact with two different ligands at the same time [44]. In that case, our construct could have only partially inactivated the hPCL3 PHD1 domain.

The two hPCL3 isoforms and particularly the short isoform hPCL3S are markedly and widely up-regulated in many types of cancers including colon, skin, lung, rectal, cervical, uterus and liver cancers, as shown by dot blot analyses of matched normal and tumors tissues from 19 different types as well as in melanoma and glioma cells lines [21].

Recent follow-up studies have finely deciphered the mechanisms implicating hPL3L/PHF9L and hPCL3S in several cancer types including glioblastomas [45], hepatocellular carcinomas [25], multiple myelomas [46], and prostate (this study). Despite the low number of studies, a clear-cut situation seems to emerge with two different mechanisms for hPCL3L/PHF19 and hPCL3S. Indeed, in close agreement with its function as a PRC2 facultative subunit [10, 14], PHF19/hPCL3L amplification is correlated with the activation of PRC2 and thus increased H3K27me deposition whereas hPCL3S is clearly involved in PRC2-independent mechanisms. In gliomas, Deng et al., reported that PHF19 is up-regulated and promotes the proliferation and migration of glioblastoma cell lines through direct repression of the promoter of *SIAH* (seven in absentia homolog 1), an E3-ubiquitin ligase of β-catenin and thus activation of the Wnt/β-catenin pathway [45]. However, the potential contribution of hPCL3S/PHF19S which is known to be expressed concomitantly with hPCL3L in several glioblastomas cell lines [21] has not been carefully investigated [45]. In a recent study, PHF19L has been clearly identified as a crucial mediator of oncogenesis in multiple myeloma through activation of PRC2 [46]. Several functional assays demonstrated that mechanistically this effect is independent of the small isoform but rely on the interaction of PHF19L with PRC2 components to facilitate the formation of broad H3K27me3-containing genomic domains, possibly through promotion of initial recruitment of PRC2 and subsequent spreading of H3K27me3 [46].

Regarding the short isoform of hPCL3/PHF19, *hPCL3S*, it has been shown to be up-regulated in hepatocellular carcinomas (HCC) clinical samples and to promote the growth and migration of HCC *in vitro* as well as their metastasis *in vivo* using mouse xenografts models. At the mechanistic level, these effects are totally independent of the PRC2 complexes but rely on the activation of the Wnt/βcatenin pathway [25]. Indeed, the cytoplasmic hPCL3S isoform has no significant effect on βcatenin mRNA transcription but rather interacts directly with cytoplasmic components of the βcatenin destruction complex, notably βTrp, the E3-ligase for βcatenin. Unfortunately, these authors did not characterize the hPCL3S domain (s) involved in these interactions. Thus, hPCL3S overexpression inhibits the degradation of βcatenin, thereby activating transcription of Wnt/βcatenin target genes such as *IL6* which is a well-characterized driver of HCC [25].

Our study showed several strong similarities with the published study on HCC [25]. Indeed, we demonstrated that *hPCL3S* positively regulated the proliferation and migration properties of DU145 and PC3 cells and that these effects are also independent of PRC2 activity as shown by the TUDOR W50A point mutant abolishing H3K36me3 which behaves as wild-type hPCL3S. However, we did not detect any significant variation in βcatenin levels upon knock-down of hPCL3S in DU145 cells (Figure 10C) nor interaction with βcatenin or the E3-ligase βTrp (Supplementary Figure 5). This apparent discrepancy would suggest that the effects on cell growth and mobility mediated by hPCL3S could rely on the activation of several different cell-specific pathways. Given these results, it would be interesting to identify proteins specifically interacting with the hPCL3 PHD1 domain trough affinity purification and mass spectrometry analyses using our LNCaP hPCL3wt stable clones.

The acquisition of resistance to AR-targeted therapy, mainly through neuroendocrine transdifferentiation is a major clinical problem for prostate cancer since it is associated with poor prognosis. Whereas LNCaP cells which express AR and PSA are similar to prostate adenocarcinomas responsive to AR-depletion therapy, PC3 cells which do not express AR nor PSA but some neuroendocrine markers (NE) are characteristic of prostatic small cell neuroendocrine carcinomas (SCNC) which are aggressive tumors not responding to hormonal therapy [3]. These two cell lines could thus be viewed as models for the neuroendocrine transdifferentiation during tumor progression in prostate cancer [2]. A key finding of our study is that *hPCL3S* is found overexpressed in hormone-independent (DU145 and PC3) but not in the hormone-dependent cell line, LNCaP. Strikingly, stable transfection of hPCL3S results in the emergence of stable clones of LNCaP cells which have acquired some characteristic of the PC3 SCNC cell line, namely a stronger proliferation and migration potential together with the loss of AR and PSA expression as well as low expression of neuroendocrine markers such as NSE and FAM184A.

In conclusion, we demonstrated that overexpression of *hPCL3S* promoted proliferation, anchorage-independent growth and migration in human LNCaP prostate cancer cells whereas silencing of endogenous *hPCL3S* in DU145 and PC3 prostate cancer cells impaired these effects. Furthermore, our results suggested that hPCL3S did not act through the perturbation of PRC2 activity but rather mainly through chromatin-independent effects relying on its PHD1 domain. Therefore, our studies could have identified the overexpression of hPCL3S as a new alteration contributing to tumor progression in prostate cancer through the emergence of a rapidly growing cell population of neuroendocrine-like cells.

## MATERIALS AND METHODS

### Patient information and tissue selection

All PCa patients included in this study had undergone radical prostatectomy in the Lille University Hospitals. Clinical data and patient consent were provided by the referring physician (Supplementary Table 1). Total RNAs used in RT-qPCR analyses were extracted from frozen tissues corresponding to matched healthy prostate and neoplastic tissue obtained from 5 patients and to primary tumors obtained from 20 other patients (age ranging from 51 to 73 years; Gleason score ranging from 6 to 9; see Supplementary Table 1 for details) as previously described [47]. These tissues were obtained from the urological collection of the local tumor tissue bank (Tumorothèque C2RC, CHU LILLE, France) after approval by the internal review board (CSTMT 225).

As control, total prostate RNAs from a 24-year donor were obtained from Biochain.

### Cell lines and cell culture

The human prostate cancer cell lines PC3, DU145 and LNCaP cells purchased from ATCC (American Type Culture Collection, Rockville, MD, USA) and the RWPE-1 cells were a kind gift of Dr. Martine Duterque-Coquillaud (CNRS UMR 8161). They were maintained in Dulbecco modified Eagle medium (Invitrogen) supplemented with 10% fetal calf serum, non-essential amino acids and gentamycin. Cells were cultured at 37°C in humidified 5% CO^2^ atmosphere as previously described [40].

For the growth in hormone-depleted medium, LNCap cells were cultured in RPMI 1640 medium (phenol red-free) supplemented with 10% of charcoal-stripped fetal bovine serum.

### Small interfering RNAs

Three siRNAs specific for hPCL3S were designed: sihPCL3S-spe1 GCTCCAAGCAGAAGGGCCA; sihPCL3S-spe2 TGGAGACAGATAGCGCCTCT and sihPCL3S-spe3 GGTTTGGTGTCG GGAATAACGG.

DU145 or PC3 cells were reverse-transfected with Lipofectamine RNAiMax (Invitrogen) according to manufacturer’s instructions using 10 nM small interfering RNA targeting *hPCL3S* or a scrambled control sequence (si Ctrl; siGENOME RISC free control siRNA, Dharmacon) [48, 49].

Forty-eight (48) hours after siRNA transfection, cells were plated and analyzed for their cell proliferation and cell migration capacities using the Incucyte technology as described below.

To verify knockdown efficiency, aliquots of these cells were treated for RNA and protein extraction and analyzed by RT-qPCR and/or Western blotting for *hPCL3S* expression levels.

EZH2-specific siRNAs were obtained from Dharmacon.

### Plasmid construction and transfection

The human *hPCL3S* ORF was PCR-amplified from the previously described pcDNA3FLAG-hPCL3S vector [22] using oligonucleotides with suitable BglII and HindIII restriction sites. The insert was cloned in the eukaryotic pAM-1C expression vector containing a puromycine selection marker (Active Motif) using the BglII site to clone it downstream of a human beta Actin promoter and the HindIII site to append the AM-tag sequence to the C-terminal end of hPCL3S. The ΔC-Term (AA 155-207) deletion mutant, the point mutant in the Tudor Domain (W50A) and the mutant (E^112^ILIC^116^ to AAQAA) inactivating an essential β-turn in the PHD1 domain were similarly cloned by a two-round PCR mutagenesis strategy. All constructs were verified by sequencing.

LNCaP cells were transfected using Lipofectamine 2000 according to the manufacturer’s instructions. At 48 hours after transfection, cells were cultured in complete selection medium containing 2 mg/ml of Puromycin. When isolated resistant clones appeared, they were recovered using the cloning cylinder method and amplified for use in all subsequent experiments.

### RNA isolation and quantitative RT-PCR analyses

Total RNA was reverse transcribed using random primers and MultiScribeTM reverse transcriptase (Applied Biosystems). Except for the primary PrEC cells (2 independent RNAs extractions), at least three independent RNA extractions (3 to 5) were analyzed for the different prostatic cell lines transfected with siRNA or with pAM-Tag vectors. Real-time PCR analysis in triplicate was performed by Power SYBR Green (Applied Biosystems) in a MX3005P fluorescence temperature cycler (Stratagene) according to the manufacturer’s instructions. Results were normalized with respect to *ALAS1* [50] or *18S* RNAs used as an internal control. The primers used for the qRT-PCR analyses are summarized in Supplementary Table 2.

### Statistics

Experiments were realized at least twice in duplicates or triplicates. Statistical analyses were performed by Student’s test. The asterix (^*^) indicates *p* < 0.05, (^**^) indicates *p* < 0.005 and (^***^) indicates *p* < 0.001. NS: Non-Significant.

### Antibodies and Western blot analyses

To generate polyclonal antibodies against hPCL3L and hPCL3S, the N-terminal common peptide corresponding to AA 1-15 of human hPCL3 (NH2-MENRALDPGTRDSYG+C-CONH2) was synthesized, coupled to KLH and used to immunize rabbits (Eurogentec, Belgium). Specific antibodies were purified by affinity chromatography using standard protocols. For hPCL3S, we also used commercial antibodies generated against a GST-hPCL3S fusion protein (Proteintech, rabbit 11895-1-AP) or against a C-terminal peptide (Everest biotech, goat EB22188).

Commercial primaries antibodies of the following specificities were also used: EZH2 (Cell Signaling, 5246), SUZ12 (Cell Signaling, D39F6), α-tubulin (Santa Cruz, sc-23948), GAPDH (Santa Cruz, sc-32223), Lamin (Santa Cruz, sc-20681), β-catenin (Santa Cruz, sc-7199), Vimentin (Santa Cruz, sc-6260), AM-Tag: (Active Motif, 61677) and normal rabbit IgG (Cell Signaling, 2729). Western blots were performed as previously described [48]. The secondary antibodies were horse-radish peroxydase-linked antibodies against rabbit, rat and mouse immunoglobulins (Amersham Biosciences) or goat immunoglobulins (Southern Biotech).

### Cell fractionation experiments

DU 145 cells grown in 100 mm plates were rinsed twice within ice-cold PBS and lysed in 1 ml of ice-cold AMNase buffer [50 mM Tris/HCl, pH7.5; 0.5% Triton X-100; 1 mMDTT and 1× protease inhibitor cocktail]. After 10 min on ice, cell lysates were centrifuged at 1800 g for 15 min at +4°C. The supernatant was recovered as the cytoplasmic fraction. The pellet was incubated for 15 min on ice in ice-cold RIPA buffer [10 mM Tris/HCl, pH7.4; 150 mM NaCl; 1 mM EDTA, 0.5% Triton X-100; 05% NaDOC, 0.1%SDS 1× protease inhibitor cocktail], briefly sonicated and centrifuged at 14 000 rpm for 15 min at +4°C. The supernatant was recovered as the nuclear fraction. Aliquots were taken (Input) and the cytoplasmic and nuclear fraction were then submitted to immunoprecipation analyses as previously described [22].

### Cell proliferation and migration assays

The cell proliferation and migration were measured using label-free and non-invasive assays by the Incucyte Live-Cell Imaging System (Essen BioScience). DU145 or PC3 cells (10.000 cells/well) were plated overnight on two 24-well plates and transfected with control or *hPCL3S*-specific siRNAs. 24 hours later, one plate was kept for 72 h before RNA extraction to check for transfection efficiency by qRT-PCR whereas the second was incubated in the Incucyte device Zoom system at 37°C in humidified 5% CO^2^ atmosphere and photographed using a × 4 objective. The proliferation has been calculated by analyzing the surface of the well (Confluence %) through live cell images collected by the Incucyte Zoom system at 2 h-intervals over 96 hours. For the migration assays, DU145 (40.000 cells) and PC3 (30.000 cells) were seeded in 96-well plates and transfected by siRNAs as described above. 24 hours after transfection, the cells were treated with Mitomycin C (10 ug/ml) for 1 hour and a wound was created using the Incucyte ZOOM 96-well Scratch wound. Then, the cells were rinsed two times by complete medium and incubated in the Incucyte Zoom system, as described above except that the cells were photographed using a × 10 objective.

For the LNCaP stably transfected clones, the same protocols were used with minor modifications. Briefly, 10.000 cells/well in 24-well plates and 35.000 cells/well in 96-well plates were used for the proliferation and migration assays, respectively. For the migration assays, the cells were treated with Mitomycin C (10 μg/ml) for 2 hours.

### Clonogenic assays

DU145 cells transfected with the relevant siRNAs and LNCaP stable clones obtained after transfection with the empty pAM vector or each hPCL3S-AMTag version were plated in 60 mm culture dishes at a density of 750 cells/dish and cultured in complete medium for 12–15 days. The medium was changed every 3 day and for the experiments using siRNAs, a second round of transfection was performed after 6 days. The colonies were rinsed with PBS before fixation with 4% PFA at 4°C and stained with 5% crystal violet for 10 min at room temperature. Then, the number of colonies was counted using the software “Colony” on a LAS3000 device.

### RNA-seq and expression analysis

Total RNA was isolated from a pool of LNCaP-pAM (empty vector) stable clones and from the LNCaP-p-AM-hPCL3S clone 12 cells using NucleoSpin RNA (Clontech). 0.5–1 μg of total RNA was treated with RiboGold zero to remove ribosomal RNA. Illumina sequencing libraries were constructed using random primers according to the manufacturer’s instructions using the TruSeq Stranded RNA LT Kit [51]. Reads were aligned to hg19 using STAR [52]. Mapped reads were filtered to exclude PCR duplicates and reads mapping to known ribosomal coordinates, from rmsk table in the UCSC database (http://genome.ucsc.edu). Gene expression was calculated using featureCounts [53]. Only primary alignments with mapping quality of 10 or more were counted. Counts were normalized to 1 million reads. Signal tracks were generated using BEDTools [54]. Differential expression was calculated using DESeq2 [55]. For gene ontology analyses, we used the DAVID database [56, 57].

## SUPPLEMENTARY MATERIALS




