# Supplementary material for: hPCL3S promotes proliferation and migration of androgen-independent prostate cancer cells
Source: Oncotarget. 2020 Mar 24;11(12):1051–74. doi: 10.18632/oncotarget.27511 (PMC7105160; doi:10.18632/oncotarget.27511)
Supplement: Supplementary file 1 [file oncotarget-11-1051-s001.pdf]

hPCL3S promotes proliferation and migration of androgen-independent prostate cancer cells

SUPPLEMENTARY MATERIALS

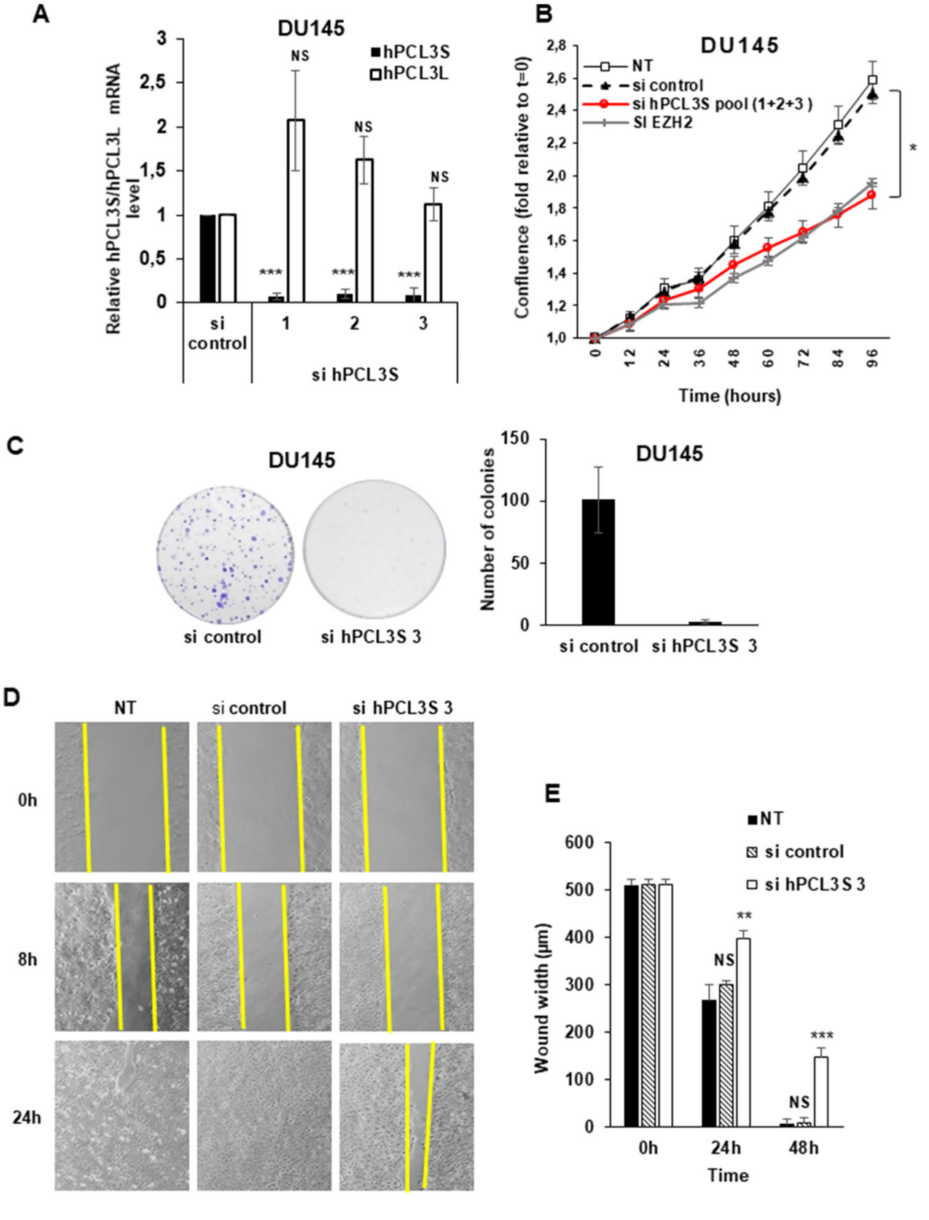

Supplementary Figure 1: The effect of *hPCL3S* knockdown in DU145 was evaluated in Wound healing and clonogenicity assays. (**A**) Validation of the three individual siRNAs targeting *hPCL3S* and analyses of their potential off-target effects on *hPCL3L* expression. After transfection of DU145 cells with the indicated siRNAs, RNAs were prepared and analyzed for the expression of *hPCL3S* and *hPCL3L* by RT-qPCR analyses. (**B**) Knockdown of *hPCL3S* inhibited the cell proliferation of DU145 cells. The proliferation of non-transfected cells and of cells transfected by a mixture of the three individual hPCL3S siRNAs instead of each individual hPCL3S-specific siRNA (see Figure 4C in the main text), was examined using the Incucyte system. Cells transfected with a control siRNA or with a siRNA targeting EZH2 were also analyzed as controls. (**C**) Clonogenicity assay. DU145 cells transfected with the control siRNA or with the *hPCL3S* siRNA 3 were compared in a clonogenicity assay (crystal blue staining). (**D**) Wound healing assays of DU145 cells transfected with the control siRNA or with the *hPCL3S* siRNA 3. The black lines correspond to the boundaries of the wound at the indicated times. (**E**) Graphical representation of the experiment shown in panel D.

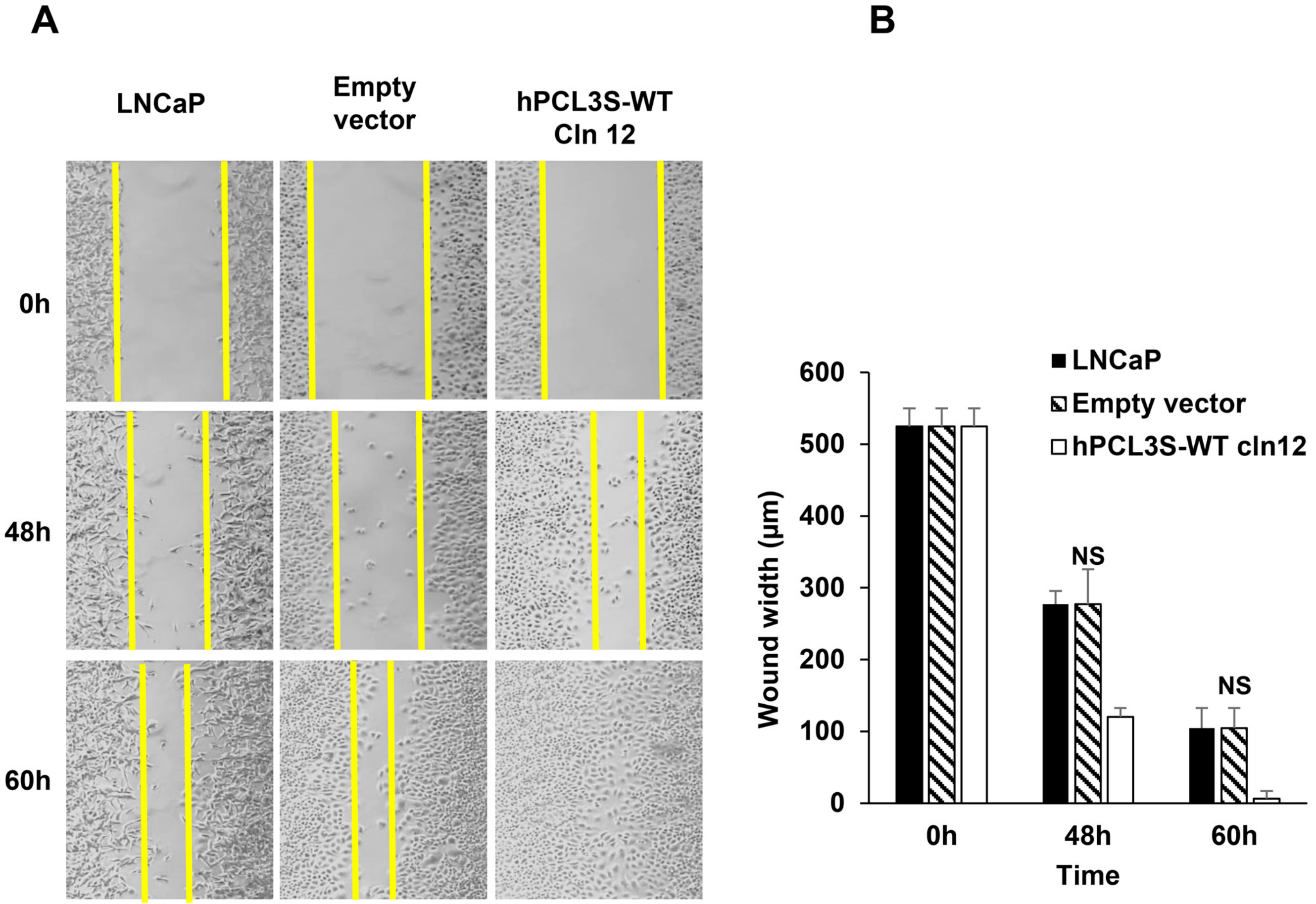

Supplementary Figure 2: The effect of *hPCL3S* overexpression in LNCaP cells was evaluated in Wound healing assays. (**A**) Wound healing assays of parental LNCaP cells, of a pool of LNCaP clones obtained after transfection with the empty vector and of the LNCaP-hPCL3S clone 12. In this preliminary experiment, the cells were cultured in complete medium without Mitomycin treatment for 60 hours (see main text for details). The black lines correspond to the boundaries of the wound at the indicated times. (**B**) Graphical representation of the experiment shown in panel A).

Empty vector | **hPCL3S- WT Cln 12** | **hPCL3S ΔC-term Cln E** | **hPCL3S ΔC-term Cln A**

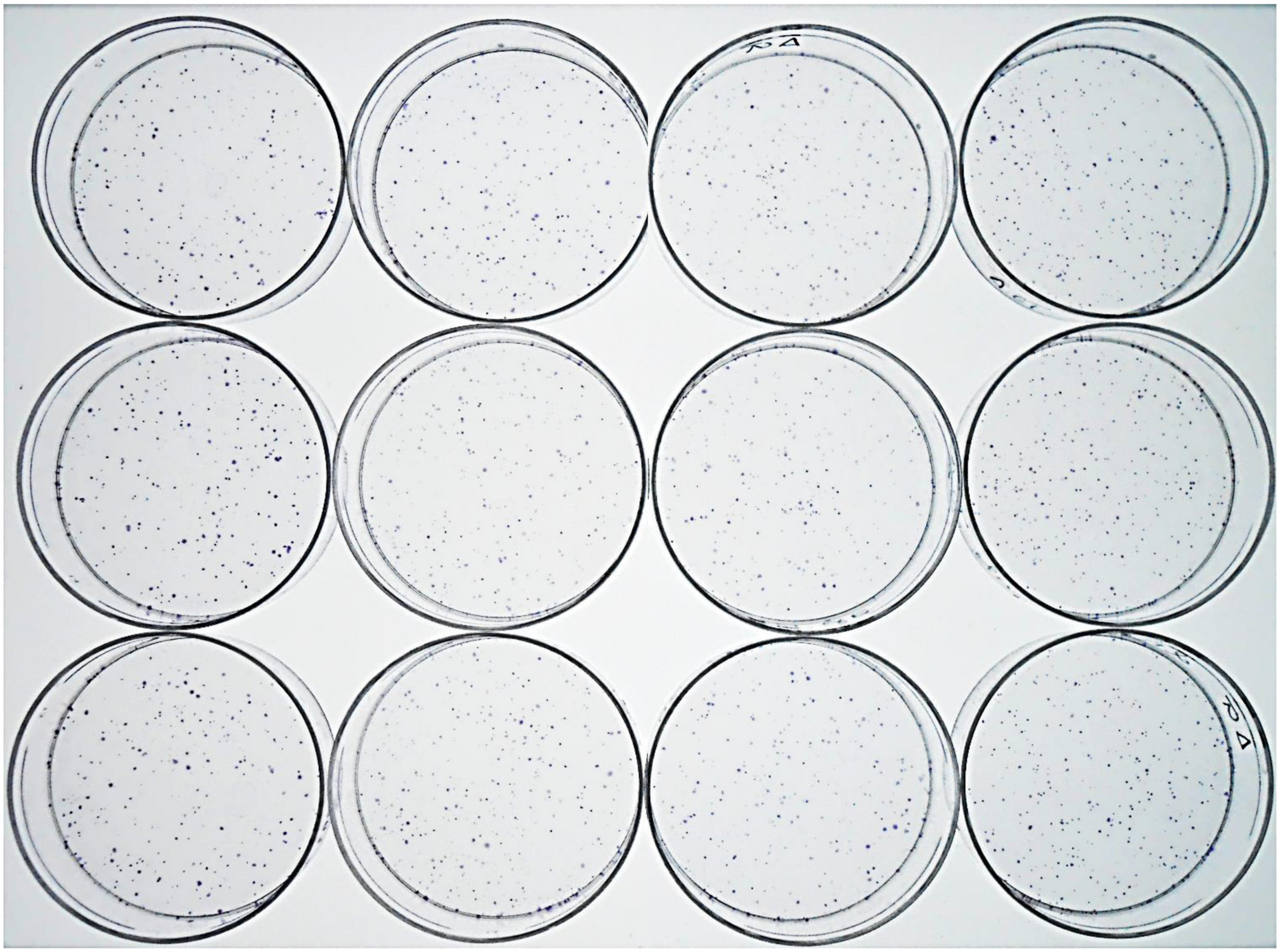

Supplementary Figure 3: The effects of wt hPCL3S and ΔC-term overexpression in LNCaP cells was evaluated in clonogenicity assays. The pictures of the plates after crystal blue staining are shown. The colonies were counted using the Colony (FujiFilm) software and the resulting graphical view is shown in Figure 7C.

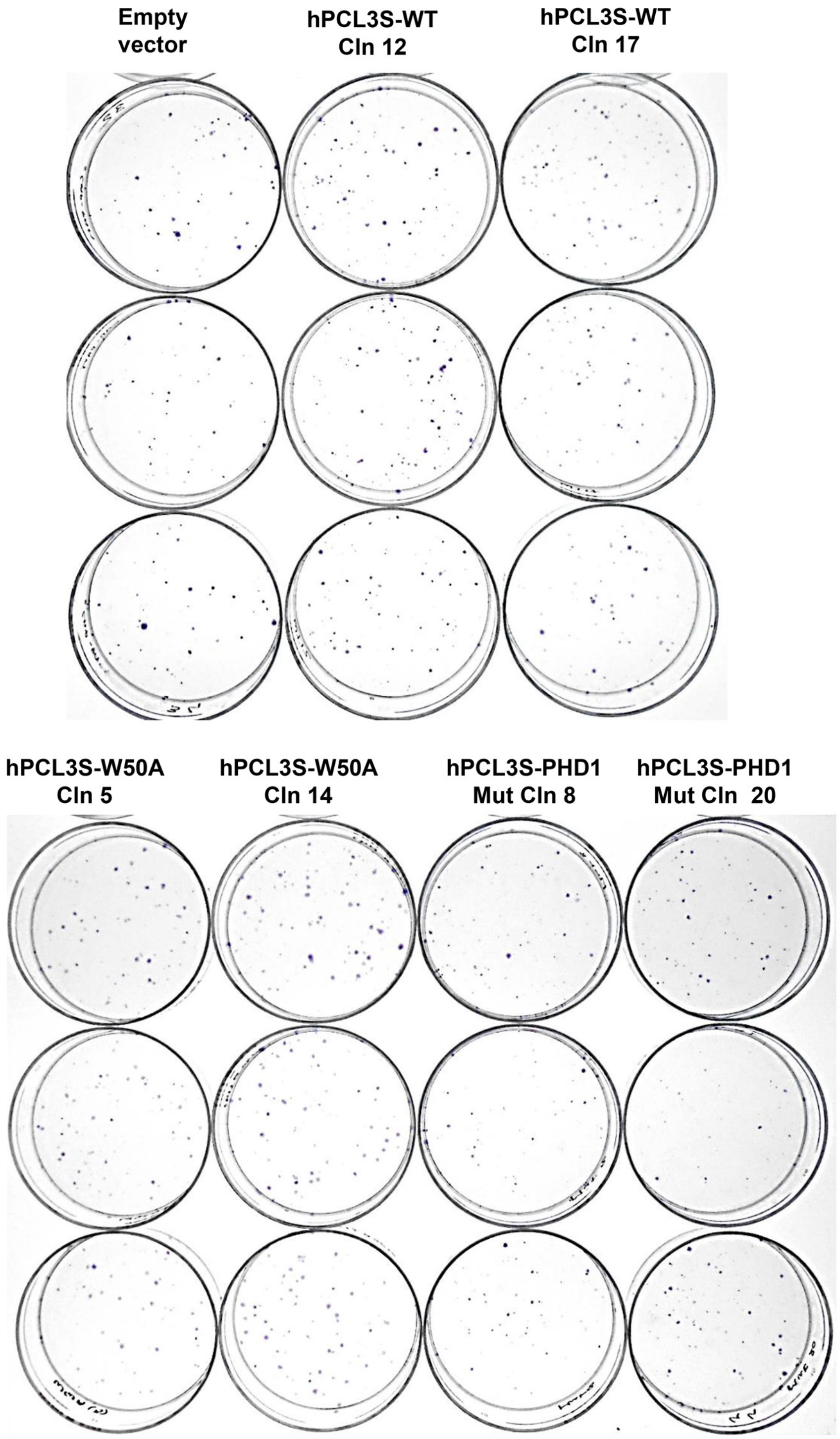

Supplementary Figure 4: The effects of wt hPCL3S, W50A and PHD1-Mut overexpression in LNCaP cells was evaluated in clonogenicity assays. The pictures of the plates after the crystal blue staining are shown. The colonies were counted using the Colony (FujiFilm) software and the resulting graphical view are shown in Figures 8C and 9C, respectively.

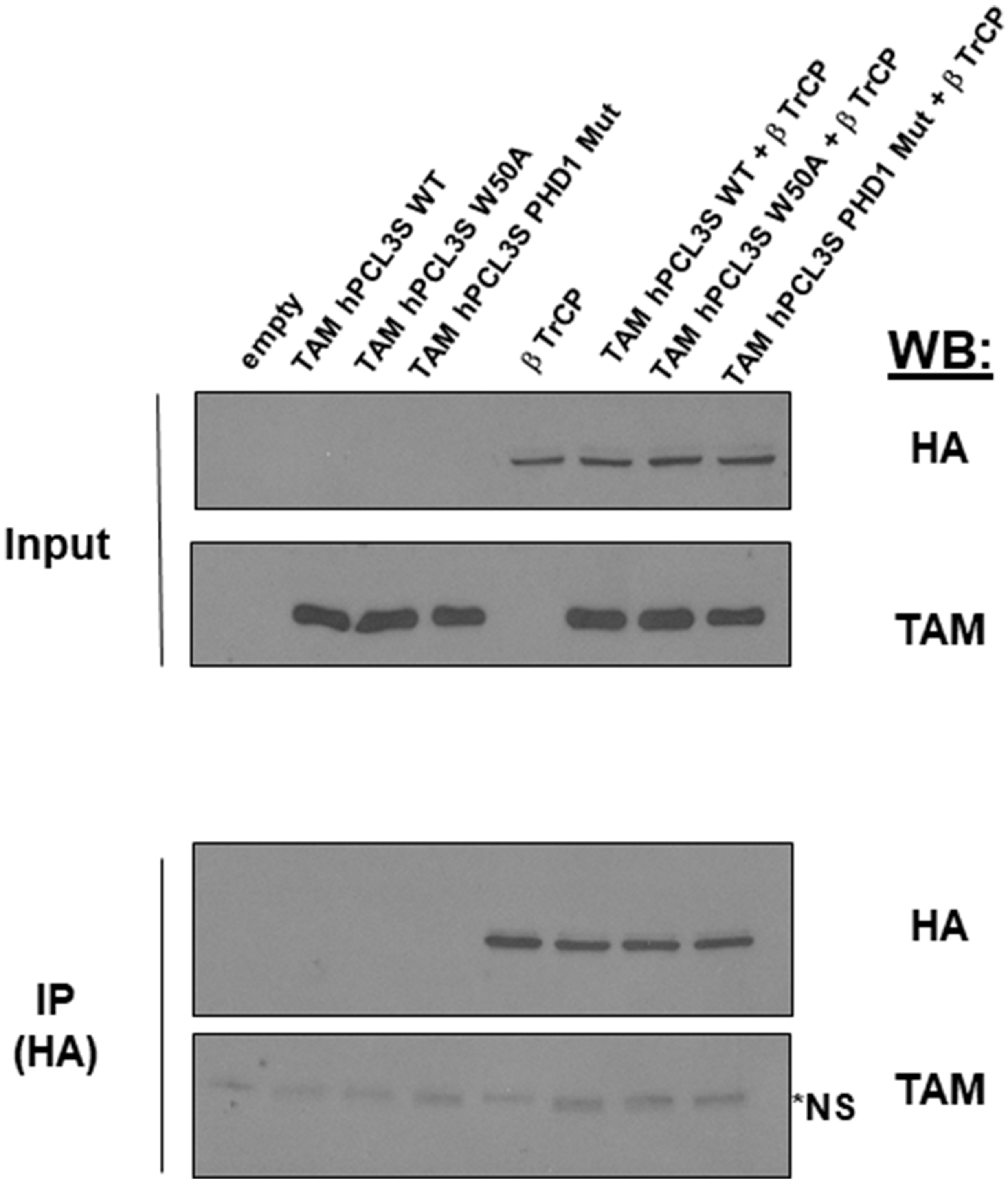

Supplementary Figure 5: hPCL3S did not interact with the E3-ligase βTRCP. HEK293T cells were transiently transfected for 48 hours with the bove-indicated combinations of expression vector for hPCL3S (wt and mutants) fused to AM-Tag and for the E3-ligase βTRPC fused to an HA epitope (plasmid pAS1B-HA-βTRC kindly provided by Dr F Margotin-Goguet, Institut Cochin, Paris). Whole cells extracts were prepared in IPH buffer (Boulay et al., 2011) and incubated with anti-HA antibodies (Bottom panels: IP (HA)) and immunoblotted with polyclonal antibodies against TAM to detect co-immunoprecipation. A non-specific signal (NS) was observed in all lanes (*). The relevant piece of the membrane was probed with anti-HA antibody to control the co-immunoprecipitation process. Furthermore, 1% of each lysate were directly resolved by SDS-PAGE and immunoblotted with the indicated antibodies to ascertain for the presence of each protein (Upper panels: Input).

```
CLUSTAL O(1.2.4) multiple sequence alignment


Otolemur garnettii          MENRALDPGTRDSYGANSHLPNKGALTKAKNNFKDLMSKLTEGQYVLCRWTDGLYYLGKI    60
Galeopterus variegatus      MENRALDPGTRDSYGATNHLPNKGALAKAKNNFKDLMSKLTEGQYVLCRWTDGLYYLGKI    60
Pan troglodytes             MENRALDPGTRDSYGATSHLPNKGALAKVKNNFKDLMSKLTEGQYVLCRWTDGLYYLGKI    60
Homo sapiens                MENRALDPGTRDSYGATSHLPNKGALAKVKNNFKDLMSKLTEGQYVLCRWTDGLYYLGKI    60
Pongo abelii                MENRALDPGTLDSYGATSHLPNKGALAKVKNNFKDLMSKLTEGQYVLCRWTDGLYYLGKI    60
Cercocebus atys             MENRALDPGTRDSYGATSHLPNKGALAKVKNNFRDLMSKLTEGQYVLCRWTDGLYYLGKI    60
Piliocolobus tephroceles    MENRALDPGTRDSYGATSHLPNKGALAKVKNNFRDLMSKLTEGQYVLCRWTDGLYYLGKI    60
Cons                        ********** *****..********:*.****:**************************

Otolemur garnettii          KRVSSSKQSCLVTFEDNSKYWVLWKDIQHAGVPGEEPKCNICLGKTSGPMNEILICGKCG    120
Galeopterus variegatus      KRVSSSKQSCLVTFEDNSKYWVLWKDIQHAGVPGEEPKCNICLGKTSGPLNEILICGKCG    120
Pan troglodytes             KRVSSSKQSCLVTFEDNSKYWVLWKDIQHAGVPGEEPKCNICLGKTSGPLNEILICGKCG    120
Homo sapiens                KRVSSSKQSCLVTFEDNSKYWVLWKDIQHAGVPGEEPKCNICLGKTSGPLNEILICGKCG    120
Pongo abelii                KRVSSSKQSCLVTFEDNSKYWVLWKDIQHAGVPGEEPKCNICLGKTSGPLNEILICGKCG    120
Cercocebus atys             KRVSSSKQSCLVTFEDNSKYWVLWKDIQHAGVPGEEPKCNICLGKTSGPLNEILICGKCG    120
Piliocolobus tephroceles    KRVSSSKQSCLVTFEDNSKYWVLWKDIQHAGVPGEEPKCNICLGKTSGPLNEILICGKCG    120
Cons                        *************************************************:**********

Otolemur garnettii          LGYHQQCHIPIAGSADRPLLTPWFCRRCIFALAVRVSPPSLPFPASLASSSGAAQRTPAP    180
Galeopterus variegatus      LGYHQQCHIPIAGGADQPLLTPWFCRRCIFALAVRVSPPSFPVTASPASSSQAAQRLPGL    180
Pan troglodytes             LGYHQQCHIPIAGSADQPLLTPWFCRRCIFALAVRVSLPSSPVPASPAFSSGADQRLP--    178
Homo sapiens                LGYHQQCHIPIAGSADQPLLTPWFCRRCIFALAVRVSLPSSPVPASPASSSGADQRLP--    178
Pongo abelii                LGYHQQCHIPIAGSADQPLLTPWFCRRCIFALAVRVSLPSSPVPASPASSSGADQRLP--    178
Cercocebus atys             LGYHQQCHIPIAGSADQPLLTPWFCRRCIFALAVRVSLSSSPVPASPASSSGAAQRLQ--    178
Piliocolobus tephroceles    LGYHQQCHIPIAGSADQPLLTPWFCRRCIFALAVRVSLSSSPVPASPASSSGAAQRLQ--    178
Cons                        *************.**:********************  * *. ** * ** * **

Otolemur garnettii          RPDFSFQSLSSMQRGHTWALGTGLPLCHLISCMSLPRLHCLPEPVKLT    228
Galeopterus variegatus      RPDSPSRNLSSKQKGHAWALRTYGASDAYQL-YVLGR---VP------    218
Pan troglodytes             -----SQSLSSKQKGHTWALETDSASATVLG-QDL-------------    207
Homo sapiens                -----SQSLSSKQKGHTWALETDSASATVLG-QDL-------------    207
Pongo abelii                -----SQSLSSKQKGHTWALETDSASATVLG-QDL-------------    207
Cercocebus atys             -----SQSLSSKQKGHTWALETDSASATVLG-QDL-------------    207
Piliocolobus tephroceles    -----SQSLSSKQKGHTWALETDSASATVFG-QDL-------------    207
Cons                              :.*** *:**:*** *            *
```

Supplementary Figure 6: The specific *hPCL3S* C-terminal coding sequence generated by the alternative polyadenylation mechanism is conserved only in 6, mostly primate, genomes. The amino-acid sequence corresponding to the hPCL3S/PHF19S specific C-terminal end (AA 155-207, highlighted in yellow) was used as a query to screen the non-redundant protein database using the BLASTP suite (https://blast.ncbi.nlm.nih.gov/Blast.cgi) to retrieve the known orthologs of hPCL3S. The results obtained included in addition to the human sequence (NP_0010009936.1) the following sequences from sumatran orangutan (Pongo abelii, XP_024107787.1); chimpanzee (Pan troglodytes, PNI69494.1); sooty mangabey (Cercocebus atyus, XP_011946756.1); ugandan red colobus (Piliocolobus tephrosceles, XP_023079511.1); small-eared galago (Otolemur garnettii, XP_023366369.1) and sunda flying lemur (Galeopterus variegatus, XP_008566995.1). The corresponding sequences were aligned using the Clustal omega multiple sequence alignment program (https://www.ebi.ac.uk) and default parameters. In the consensus lane, (Cons) identical residues are shown as *; conserved substitutions as : and semi-conserved substitutions as . under the aligned sequences.

Supplementary Table 1: Clinical/pathological characteristics of the prostate tumors

	Localisation	Age at collection	total PSA	Gleason	TNM	Morphological control: % Tumor	% normal tissue	% tumor necrosis	% tumor cells	% Stroma
1	Normal	63 years	8 ng/ml			0	100	0		
	Tumor			3+3=6	pT2c	80	20	0	60	40
2	Normal	66 years	9,77 ng/ml			0	100			
	Tumor			3+4=7	pT3a	90	10	0	70	30
3	Normal	67 years	5,4 ng/ml			0	100			
	Tumor			3+4=7	unknown	80	20	0	40	60
4	Normal	67 years	9,65 ng/ml			0	100			
	Tumor			4+3=7	pT2c	70	25	0	80	20
5	Normal	57 years	6,2 ng/ml			0	100			
	Tumor			3+4=7	pT2c	60	40	0	70	30
6	Tumor	65 years	11,8 ng/ml	7 (3+4)	pT2b	100	0	0	80	20
7	Tumor	73 years	6,73 ng/ml	9 (4+5)	pT3b	80	20	0	70	30
8	Tumor	52 years	6,86 ng/ml	9 (4+5)	pT2x	80	20	0	80	20
9	Tumor	69 years	5 ng/ml	7 (3+4)	pT3a	80	20	0	80	20
10	Tumor	69 years	13,9 ng/ml	7 (3+4)	pT2c	95	5	0	80	20
11	Tumor	59 years	10,25 ng/ml	7 (4+3)	pT3b	100	0	0	80	20
12	Tumor	72 years	6,92 ng/ml	7 (4+3)	pT3a	95	5	0	80	20
13	Tumor	68 years	32,4 ng/ml	9 (4+5)	pT3b	90	10	0	90	10
14	Tumor	56 years	22,3 ng/ml	7 (4+3)	pT3a	90	10	0	90	10
15	Tumor	68 years	20 ng/ml	9 (4+5)	pT2c	90	10	0	90	10
16	Tumor	51 years	14,3 ng/ml	8 (4+4)	pT3a	80	20	0	80	20
17	Tumor	59 years	6,91 ng/ml	7 (4+3)	pT2b	95	5	0	90	10
18	Tumor	69 years	7,75 ng/ml	7 (4+3)	pT3b	80	20	0	80	20
19	Tumor	60 years	7,07 ng/ml	7 (4+3)	pT3a	95	5	0	95	5
20	Tumor	68 years	5,6 ng/ml	7 (4+3)	pT3b	70	30	0	60	40
21	Tumor	58 years	5,07 ng/ml	9 (4+5)	pT3a	90	10	0	90	10
22	Tumor	69 years	9 ng/ml	9 (4+5)	pT3a	90	10	0	80	20
23	Tumor	72 years	7 ng/ml	9 (4+5)	pT3b	90	10	0	80	20
24	Tumor	59 years	4,9 ng/ml	7 (3+4)	pT3b	80	20	0	80	20
25	Tumor	64 years	6,15 ng/ml	7	pT2a	80	20	0	80	20

Supplementary Table 2: Oligonucleotides used in RT-PCR analyses

**hPCL3L**	sense	GTTTGGAGACCGGTTTTACCT
**hPCL3L**	anti sense	CTTGCTCTGTACCCCCAGATTATA
**hPCL3S**	sense	CCTCATCTGCGGGAAGTGT
**hPCL3S**	anti sense	CAGGAGAGGCAGGGACTG
**18S**	sense	GGCGCCCCCTCGATGCTCTTAG
**18S**	anti sense	GCTCGGGCCTGCTTTGAACACTCT
**EZH2**	sense	TGCAGTTGCTTCAGTACCCATAAT
**EZH2**	anti sense	ATCCCCGTGTACTTTCCCATCATAAT
**ALAS1**	sense	TGGTGCAGTAATGACTACCTAGGA
**ALAS1**	anti sense	CCCCAGCACCATGTTGTTT
**NSE**	sense	GAGACAAACAGCGTTACTTAG
**NSE**	anti sense	AGCTGCCCCTGCCTTAC
**IL-6**	sense	TTCGGTCCAGTTGCCTTCTC
**IL-6**	anti sense	TACATGTCTCCTTTCTCAGGGC
**S100A16**	sense	ATGCTGTCGGACACAGGGAA
**S100A16**	anti sense	TGATGCCGCCTATCAAGGTC
**FAM 184 A**	sense	TGGAGGGCTTCCGGATAGAA
**FAM 184 A**	anti sense	GCAAGGAAGCAAGCATAGCC
**PLXNA2**	sense	GGAGCCACTCTTCATGCTATACTGT
**PLXNA2**	anti sense	GTTGACGCAGTTCAGGATCA
**SPON1**	sense	ACAGCGGATGCTCAAGTCTC
**SPON1**	anti sense	AGTCAATGGGGCATTCAGGG
**AR**	sense	TGAAGCTTCTGGGTGTCACT
**AR**	anti sense	CTTCTGTTTCCCTTCAGCGG
**PSA**	sense	AGTGCGAGAAGCATTCCCAAC
**PSA**	anti sense	CCAGCAAGATCACGCTTTTGTT
**ALDHA1**	sense	GGAACAGTGTGGGTGAATTGC
**ALDHA1**	anti sense	GGAAACCGTACTCTCCCAGT

Supplementary Table 3: Differentialy regulated genes. See Supplementary Table 3
